# Designing Chimeric Molecules for Drug Discovery by Leveraging Chemical Biology

**DOI:** 10.1021/acs.jmedchem.9b01456

**Published:** 2020-02-05

**Authors:** Chiara Borsari, Darci J. Trader, Annalisa Tait, Maria P. Costi

**Affiliations:** † Department of Biomedicine, University of Basel, Mattenstrasse 28, 4058 Basel, Switzerland; ‡ Department of Medicinal Chemistry and Molecular Pharmacology, Purdue University, 610 Purdue Mall, West Lafayette, Indiana 47907, United States; § Department of Life Sciences, University of Modena and Reggio Emilia, Via Campi 103, 41125 Modena, Italy

## Abstract

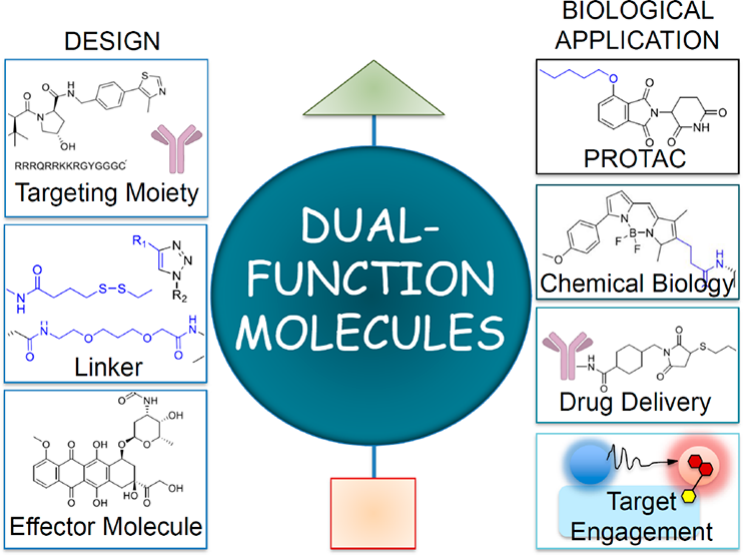

After the first seed concept introduced in the 18th century, different disciplines have attributed different names to dual-functional molecules depending on their application, including bioconjugates, bifunctional compounds, multitargeting molecules, chimeras, hybrids, engineered compounds. However, these engineered constructs share a general structure: a first component that targets a specific cell and a second component that exerts the pharmacological activity. A stable or cleavable linker connects the two modules of a chimera. Herein, we discuss the recent advances in the rapidly expanding field of chimeric molecules leveraging chemical biology concepts. This Perspective is focused on bifunctional compounds in which one component is a lead compound or a drug. In detail, we discuss chemical features of chimeric molecules and their use for targeted delivery and for target engagement studies.

## 1 Introduction

A chimeric molecule is an engineered construct in which two or more components are linked to form a novel biological agent. Chimeric molecules can be considered as variants of an idea proposed by Paul Ehrlich in the late 1800s. This concept describes a bifunctional molecule in which one component targets the molecule to a specific cell and the second component exerts a pharmacological activity.^1,2^ Different disciplines have attributed multiple names to dual-functional molecules (chimeras, hybrids, bioconjugates, bifunctional compounds, multitargeting molecules, engineered compounds) depending on the field of application, but the general structure is conserved.

**Figure 1:**
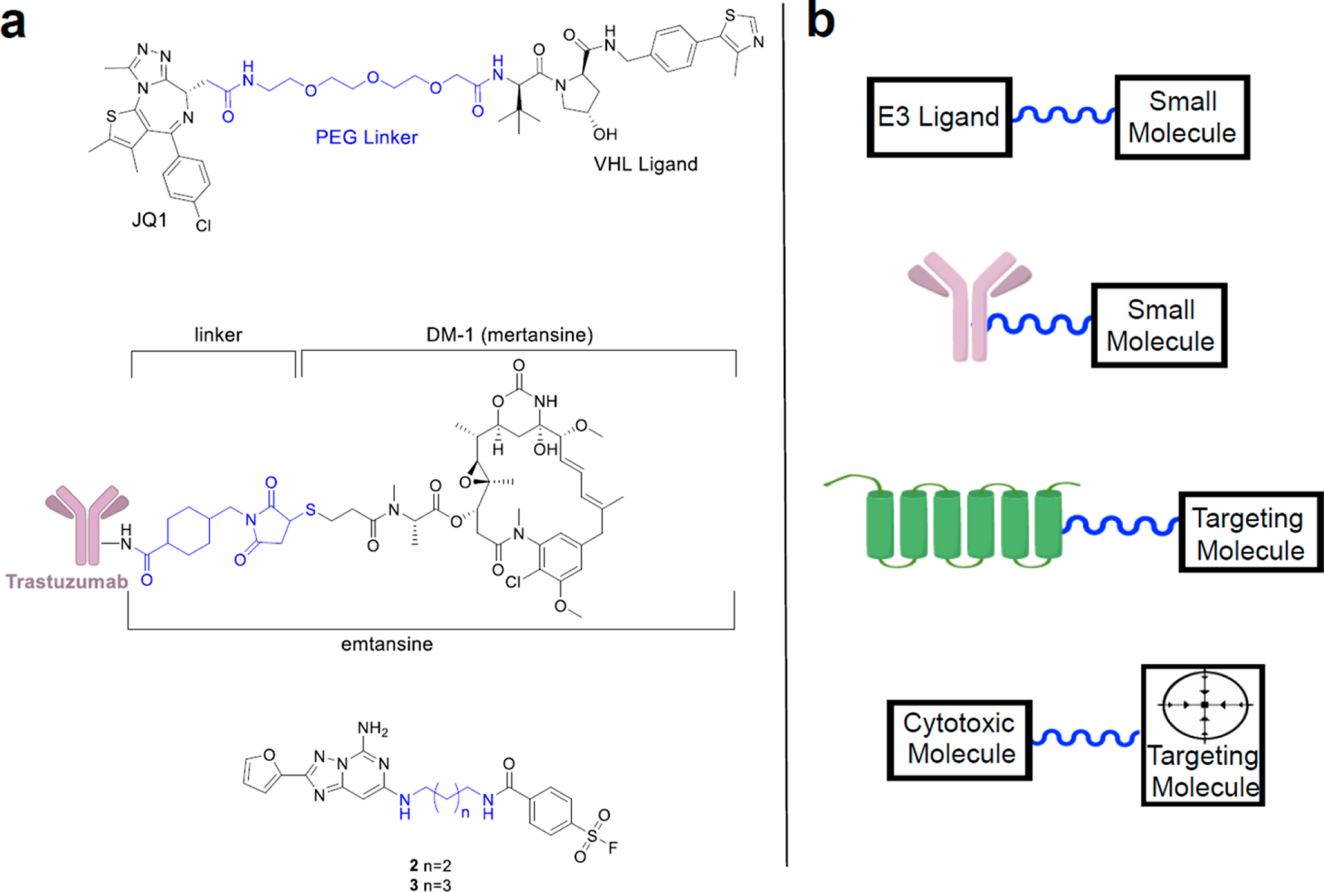
Overview of chimeric compounds with a diversity of structures: (a) examples of chimeras discussed in the Perspective, where linker moiety is highlighted in blue; (b) general structures of chimeric compounds.

Recently, the knowledge in cellular and molecular biology widely increased. The chemical biology field allowed the application of the chemistry knowledge to deliver specific biomolecules on the cell membrane and into the cells. The concepts of chemical biology were translated into drug discovery of chimeric molecules (or chimeras).^3,4^ These entities display (i) a targeting moiety and (ii) an effector molecule within the same chemical construct, and their individual function could be largely modulated with appropriate conjugation chemistry strategies where a linker is the bridging element (Figure 1).^1,3^ Recently, the exploitation of these systems for drug delivery implementation, particularly into cancer cells, has been reviewed.^4^

This Perspective discusses the recent advances in the rapidly expanding field of chimeric molecules in which one component is a lead compound or a drug. In detail we discuss chemical features of chimeric molecules, targeted delivery, and the exploitation of chimeric molecules for target engagement studies.

Section 2 is focused on linker chemistry. To develop small molecules that engage a specific cell type or protein target, a small molecule needs to be linked with another moiety that allows selective target recognition. The linker plays a pivotal role in the development of chimeric compounds and allows bridging of two pharmacophores within one molecule. The type and the length of the linker are essential parameters for the design and biological activity of chimeras, leading to a rapid expansion of the linker chemistry field.

Section 3 deals with drug delivery based on receptor-mediated endocytosis (RME). Cell membrane permeation represents the major bottleneck in achieving the sufficient drug concentration for therapeutic effect. Drug delivery systems exploiting receptor-mediated endocytosis have been proposed as a promising tool to overcome tissue barriers and have given an important contribution to medical practice, especially in the area of cancer and central nervous system (CNS) disorders. Three classes of ligands have been used to target receptors at the cell membrane and are herein discussed: (i) cell-penetrating peptides (CPPs), (ii) tumor homing peptides, and (iii) monoclonal antibodies.

Section 4 covers the recent advancements in chimeric molecules engineered to demonstrate how a drug engages its own target intracellularly. Herein, we discuss the crucial integration of chemical biology knowledge, drug discovery strategies, and medicinal chemistry to foster structure–mechanism of action studies and subsequent structural modifications.

## 2 Linker Features in the Modular Approach to Chimeric Compounds

### 2.1 Linker Chemistry

Physically connecting two chemical moieties or a small molecule with a protein occurs through a moiety called linker. A wide variety of linkers have been developed that consider if the target of the small molecule is intra- or extracellular and what type of cell or tissue the small molecule needs to target. If the desired target is intracellular, typically the linker includes a moiety that can be cleaved once the chimera is inside the cell. Linkers also play an important role in activity-based protein profiling experiments.

**Figure 2:**
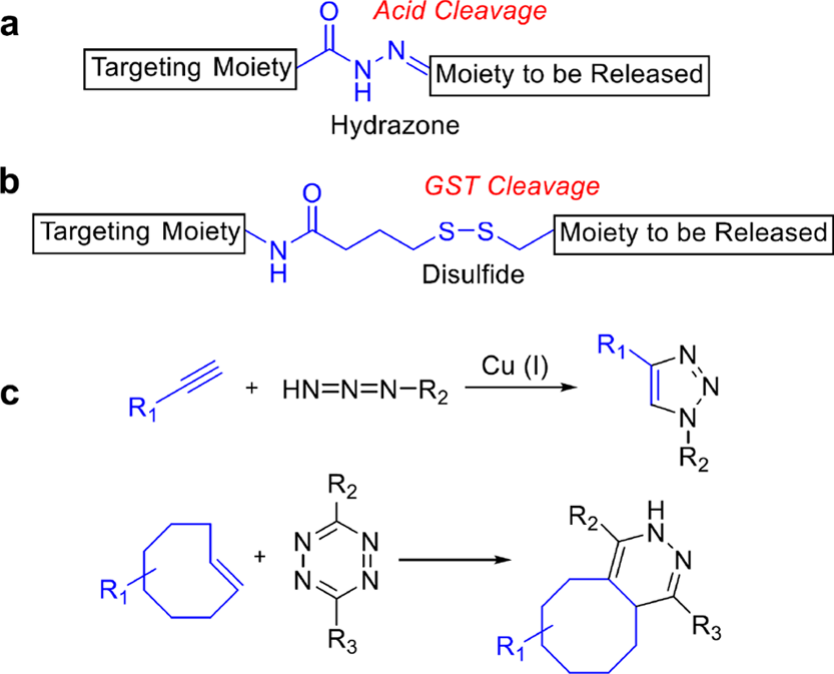
Examples of linkers and cleavage conditions: (a) hydrazone; (b) disulfide linker; (c) traditional click chemistry reaction used to easily link moieties together. The inverse Diels–Alder reaction has been used to link molecules to a solid surface for screening or for the release of a cytotoxic moiety. Linkers are highlighted in blue.

A commonly used linker type is hydrazone, Figure 2a. The hydrazone moiety can typically be easily installed because of its compatibility with peptide synthesis.^5^ The hydrazone moiety is stable at physiological pH and cleaves at an acidic pH, but additional conditions that do not require acids have been developed.^6^ While the hydrazone moiety has been widely used in diversity-oriented synthesis^7^ and as an additional handle in peptide synthesis,^8^ more recently it has been exploited as a reversible linker for proteomics experiments.^9^ Several different types have been developed, including an acyl hydrazone from the Kohn laboratory.^10^ Their study highlights a more efficient capture and release of the targeted protein pool as compared to standard protocols due to the mild conditions for the hydrazone release. Captured proteins do not have to be exposed to SDS or 8 M guanidine to release them for mass spectrometry experiments. The Dawson group developed a bisaryl hydrazone linker also highlighting the mild conditions that can be exploited to release captured proteins from a hydrazone-linked molecule.^11^

The disulfide moiety is also commonly investigated in chemical biology (Figure 2b). It has been highly utilized to cyclize peptides. The cyclization of peptides has been shown to increase their resistance to proteases and, in some cases, stabilizes the structure to boost its ability to bind to the targeted protein.^12^ More recent trends have promoted the concept of peptide stapling rather than disulfide bonds because of the reversibility of the disulfide bond.^13^ Nonetheless, disulfides in peptide therapeutics are still common, with the most well-known therapeutic peptide that incorporates disulfide bonds being insulin.^14^ Disulfide linkers have also been exploited in recombinant fusion proteins^15^ and for the synthesis of peptide libraries.^16,17^

Since the disulfide linker bond can be reversed under physiological conditions, it has been integrated into drug delivery approaches and in prodrug scaffolds. Cells have a high level of free −SH moieties in their cytosol. Once the disulfide-linked drug enters the cytosol, the disulfide can be reduced, releasing the drug moiety.^18^ The disulfide linker has been extensively used in the conjugation of small molecules to antibodies. Anticancer drugs, including doxorubicin (**1**), methotrexate, and mitomycin C, have been linked to antibodies and, after internalization, the disulfide linker is cleaved and the cytotoxic agent released.^19^ This method increases the uptake of the cytotoxic drug by cancer cells and not by the healthy ones. A linker containing a free thiol is conjugated to the small molecule of interest at a location that does not affect its activity. This entire moiety is then bound to an antibody through generation of a disulfide bond between the free thiol linked to the small molecule and a cysteine residue on the antibody.

Other linkers used in chemical biology can be generated through the reaction commonly referred to as click chemistry (Figure 2c). The term click chemistry, coined by Karl Barry Sharpless, refers to a variety of reactions that are considered simple and regiospecific and provide high yields.^20^ However, click chemistry has become traditionally referred to as the Huisgen 1,3-dipolar cycloaddition of azides and terminal alkynes. The most basic click reaction, with cooper as a catalyst, produces a 1,4-subsituted triazole. This reaction has been used to (i) link natural products to tags aiding in identification and detection,^21,22^ (ii) introduce a biotin moiety on proteins of interest for enrichment for mass spectrometry experiments,^23−26^ and (iii) synthesize a variety of small molecule libraries on solid-phase or polymer-like structures.^27−30^ A click reaction generating a releasable linker is the inverse-electron-demand Diels–Alder between a conjugated trans-cyclooctene and a tetrazine moiety. This type of cleavage linker has been demonstrated to effectively release **1** or other ligands conjugated to an antibody.^31,32^ The Garner laboratory has also employed this type of click reaction to develop different platforms for the screening of small molecule binders to RNA.^33,34^

The linkers described here are just a few of those that have been developed to help answer a variety of chemical biology questions and for therapeutic application. In the remaining subsections, we will describe more specific examples of how linkers are critical for the success of drug discovery programs and for the study of essential cellular processes.

### 2.2 PROTAC Linker Considerations

**Figure 3:**
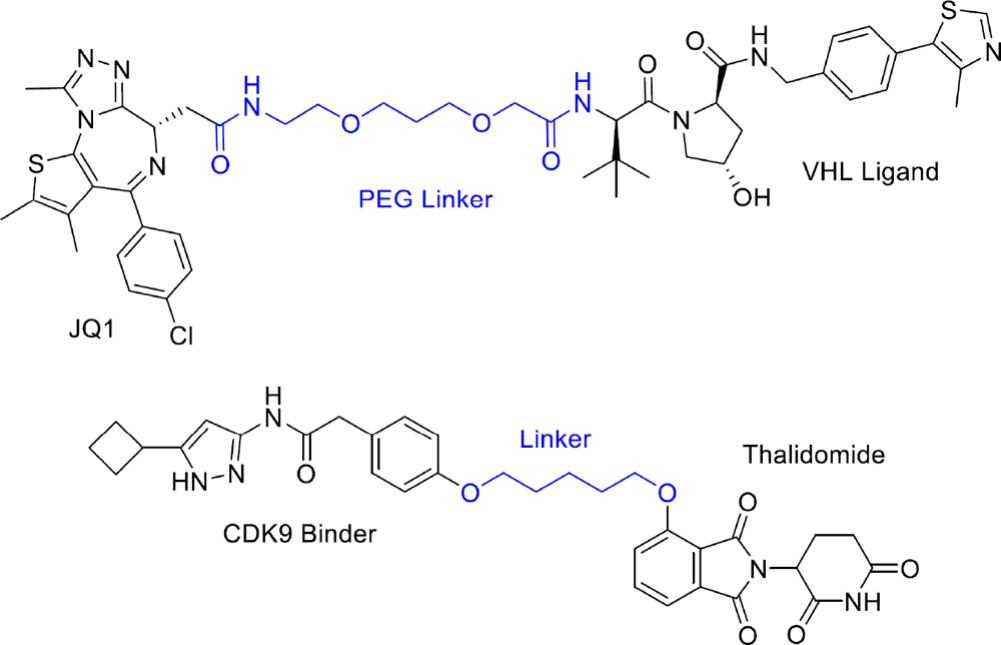
Chemical structures of the well-known, potent PROTACs including JQ1 and a CDK9 inhibitor, presenting the best linker length. Linkers are highlighted in blue.

Proteolysis targeting chimeras or PROTACs represent a new method to target proteins of interest and degrade them to elicit a therapeutic response. This method exploits a chimeric molecule. A small molecule binder to an E3 ligase is linked to another small molecule that binds with the protein of interest. The targeted protein is then ubiquitinated after coming into close contact with the E3 ligase and degraded by the proteasome. One of the most critical decisions in designing a PROTAC is the length of the linker required to connect the small molecule binding to the protein of interest and the desired E3 ligase. PROTACs have been developed to degrade a variety of target proteins including ALK,^35^ the estrogen receptor,^36^ MDM2,^37,38^ tau,^39^ BET protein, and CDK9 protein. For these two last ones, the chimeric compounds JQ-1 and CDK9 are reported in Figure 3. Well-established PROTACs are commercially available. After selection of which E3 ligase to target, typically either cereblon (CRBN) or von Hippel–Lindau (VHL), an appropriate linker between the E3 ligase binding moiety and the molecule binding the protein to be degraded needs to be installed.

An interesting study by the Kim group highlights how critical the linker length is in order to generate a potent degrader. They created an estrogen receptor (ER)-α-targeting PROTAC and installed a variety of linkers with different lengths. These linkers were composed of polyethylene glycol units, ranging in length from 11 to 16 atoms. Their results showed that while the 12- and 16-atom linkers had similar binding affinities to the ER, the 16-atom linker was significantly more potent in degrading the ER.^40^

The importance of the linker length for a PROTAC was also demonstrated by the Krönke group.^41^ They designed a homo-PROTAC for the degradation of the E3 ligase CRBN. If CRBN cannot ligate its cellular substrates, ubiquitinated proteins can increase, leading to cell death. They connected two thalidomide moieties with PEG linkers of various lengths and determined their abilities to degrade CRBN. In this case, the optimized linker was a short 8 atoms length PEG. These studies, along with many others, highlight that new PROTACs must be tested with a variety of different length linkers.^42^ Linker dynamics, such as thermodynamics, linker flexibility, and decreasing steric clash, have been studied, and all of these parameters should be considered when designing a new PROTAC.^43,44^

### 2.3 Linkers for the Discovery and Isolation of Natural Products

**Figure 4:**
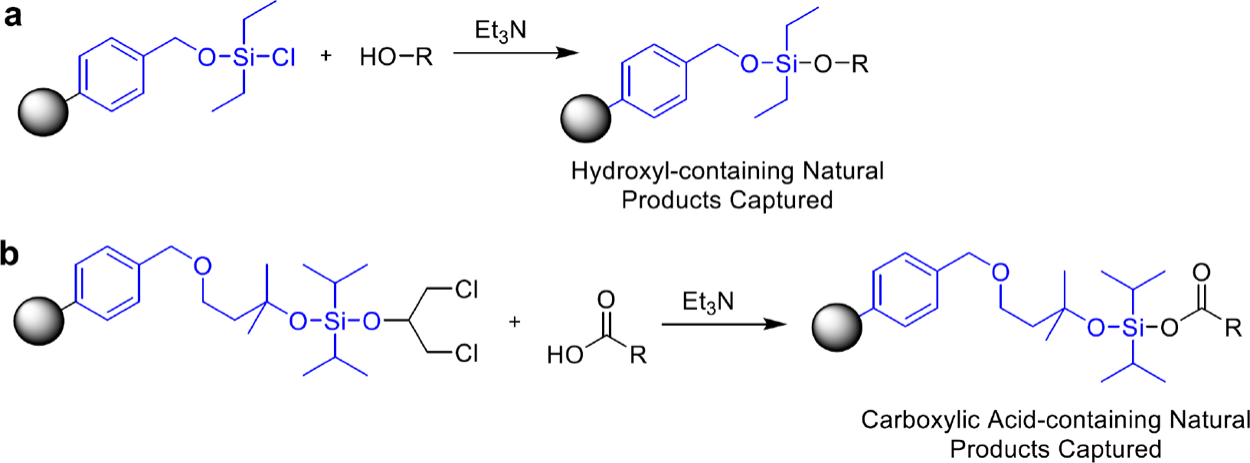
Linkers for the physical capture of natural products. Resins utilize a linker (colored in blue) with a silicon atom to capture (a) hydroxyl- or (b) carboxylic acid-containing molecules.

Natural products represent a novel pool of potential antibiotic and anticancer molecules. Traditional purification techniques are biased toward discovering natural products that have been already identified. As described in the click chemistry section, it is a method to target alkyne-containing natural products, but these are a very small pool of natural products. The biggest challenge in discovering therapeutically relevant natural products is finding small molecules that have not been previously identified. Traditional extraction methods of a crude natural product lysate followed by LC/MS analysis is biased toward discovering the most abundant molecules in the lysate. Linkers that can isolate natural products based on their functional group composition have been developed. This technique produces different pools of natural products, helping to unmask those that are too low in abundance to be detected in the crude lysate. The Carlson group has developed a family of reversible linkers to isolate hydroxyl-, phenol-, and carboxylic acid-containing natural products.^45−48^ These linkers contain different siloxy moieties that can selectively capture or release different molecules containing the aforementioned functional groups (Figure 4). The capture of hydroxyl-containing natural products occurs by the formation of a silyl ether bond. This links the natural product to the resin. Molecules not bound to the resin are rinsed away using a variety of solvents. The molecules linked to the resin can be released by exposing the resin to a fluoride source, such as TBAF or HF. This creates two pools of molecules, those that contain a hydroxyl moiety and those not bearing this functional group. These unique pools of molecules can be concentrated and analyzed by LC/MS or fractionated for activity-based assays.

Linkers for the isolation of natural products containing other functional groups have also been developed. There are several examples of linkers to capture thiol containing natural products through either a disulfide bond formation or a 1,4-nucleophilic addition.^49,50^ Linkers to natural products containing less prevalent functional groups, including epoxides and β-lactams, have also been described.^51,52^

### 2.4 Linking Covalent Inhibitors to Fluorophores

**Figure 5:**
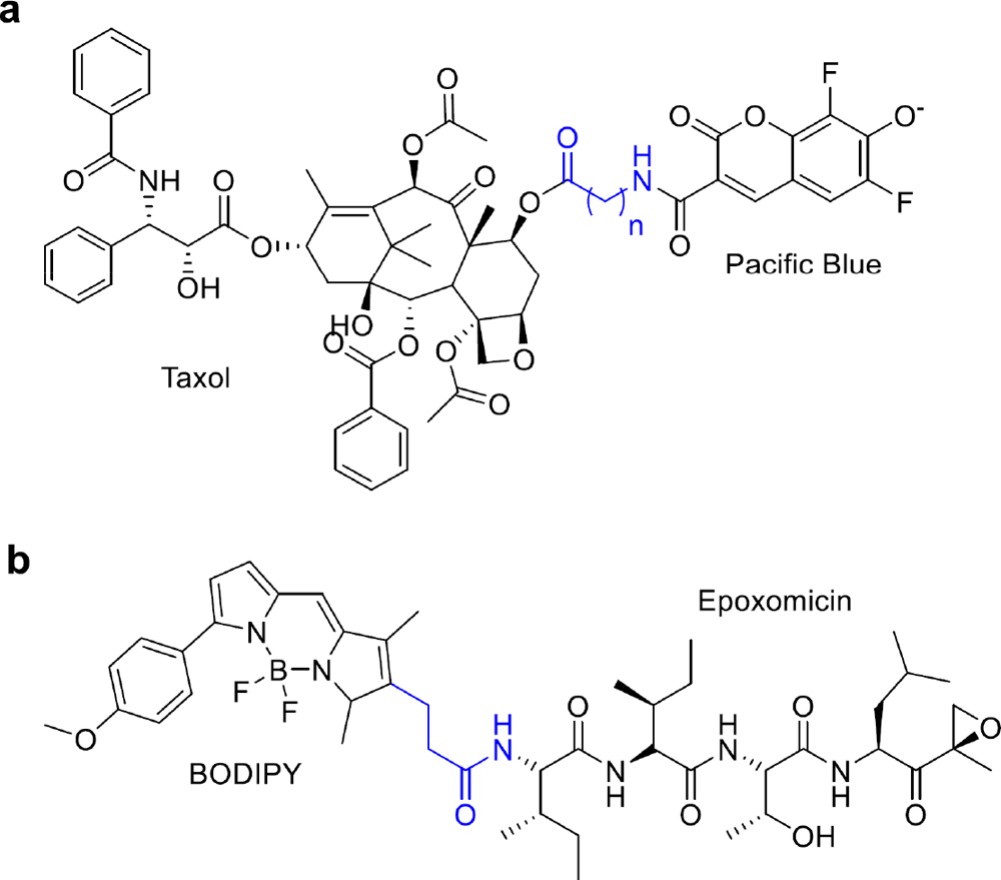
Two probes to observe (a) tubulin dynamics and (b) proteasome activity with the linker portion colored in blue. A variety of linker lengths between the small molecule binder/inhibitor and the fluorophore were evaluated to ensure that the fluorophore did not interfere with the binding to the protein of interest.

Monitoring and visualizing essential cell processes are critical for drug development. The monitoring of enzymes critical to cell survival is an important chemical biology technique. To accomplish this, a number of covalent inhibitors have been linked to fluorophores through a variety of linkers aiming to visualize the desired cellular process. These probes can be used in confocal microscopy and/or flow cytometry to evaluate the effect of potential small molecule therapeutics. One example is the development of a fluorescent derivative of Taxol (**2**) (Figure 5a). This cytotoxic drug was discovered ∼50 years ago and has been used to treat a variety of cancer types. **2** targets cells that are rapidly dividing by interacting with microtubules and initiating mitotic arrest. However, it is currently unclear the mechanism by which **2** elicits its toxic effect and why some patients do not respond to the treatment.^53,54^ To visualize the subcellular localization of **2**, the Peterson group synthesized a probe that links this microtubule-stabilizing drug to Pacific Blue.^55^ They tested three different linker lengths between **2** and the Pacific Blue moiety, and their results indicated that having a glycine linker, rather than a β-alanine or GABA linker, led to the best binding affinity to the tubulin heterodimer. Their probe was highly specific for tubulin binding, and they proposed that it can be used as a new tool for studying how **2** affects the proliferation rate of cancer cells.

Fluorescent probes with a variety of linker types have also been developed to monitor the activity of the proteasome. The proteasome is a large protein complex in cells, responsible for proteins degradation. If unwanted proteins accumulate in cells, this can lead to endoplasmic reticulum stress and eventually apoptosis.^56−58^ Fluorescent probes have been developed to study the activity of the proteasome in cells.^59,60^ One of the major considerations when developing a proteasome activity probe is the linker length between the fluorophore and the active-site binding moiety. The fluorophore must be far enough from the binding site moiety to prevent any steric hindrance but not too bulky that it cannot enter the catalytic channel of the proteasome. The Overkleeft group has developed a number of fluorescent probes to monitor the activity of the proteasome. They have applied an activity probe that consists of the BODIPY fluorophore linked to epoxomicin, a covalent inhibitor of the proteasome (Figure 5b).^61^ This probe, along with others with different linker lengths, can be used to evaluate proteasome activity and determine the composition of the different types of active sites that assemble to form the full proteasome.^62,63^ In addition, a variety of probes with different types of linkers have been developed to monitor the activity of the immunoproteasome.^64^ The immunoproteasome rather than the standard proteasome is produced when cells encounter an inflammatory signal.

The recent advancements in linker chemistry suggest that in the future linkers will allow making steps forward in the design of chimeras. Moreover, the linker will play a pivotal role in the delivery and release of therapeutic agents, as well as in the investigation of biological pathways.

## 3 Chimeric Compounds and Receptor-Mediated Endocytosis

### 3.1 Receptor-Mediated Endocytosis for Drug Delivery

**Figure 6:**
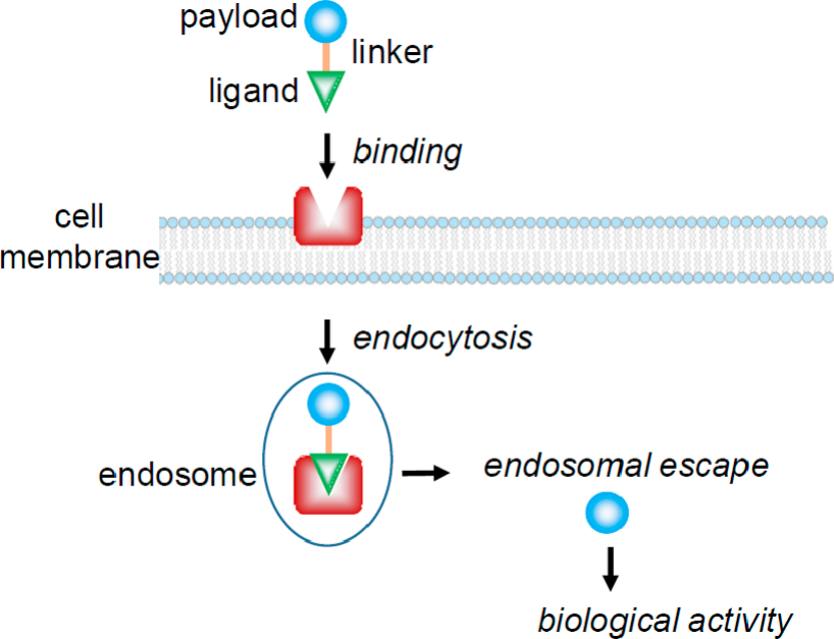
Schematic strategy of the selective delivery of biological cargos into cells exploiting receptor-mediated endocytosis.

Lack of optimal pharmacokinetic profile is one of the main reasons why compounds fail during preclinical and clinical studies. Barrier permeability is an obstacle in achieving the therapeutic effect. Drug delivery opportunities are currently rising, and researchers are focusing their work on overcoming tissue barriers. Receptor-mediated endocytosis (RME, Figure 6) has been extensively studied as a method for boosting the transport of bioactive cargo across membranes, including the blood–brain barrier (BBB).

Ligands binding to surface receptors can induce cellular uptake of therapeutics, including monoclonal antibodies, peptides, nucleic acids, small-molecule drugs, and nanoparticles. Herein, we discuss the recent advancements in the use of ligands for selectively binding to cell surface receptors. Three classes of ligands are discussed: (i) cell-penetrating peptides (CPPs), (ii) tumor homing peptides (THPs), and (iii) monoclonal antibodies. The highest ligand selectivity is displayed for antibodies, and this led to the FDA approval of five antibody–drug conjugates (ADCs): Mylotarg (**3**),^65,66^ Besponsa (**4**),^67^ Adcetris (**5**),^68,69^ Kadcyla (**6**),^70,71^ and Polivy (**7**).^72^

However, huge advancements have been shown also in the field of THPs. THPs are short peptides that have an inherent property to recognize tumor cells. Tumor necrosis factor α (hTNFα) was conjugated with a tumor homing peptide (NGR),^73,74^ and phase I and phase II clinical trials of NGR-hTNFα as a single agent and in combination with **1** are ongoing. In addition, THPs have a possible application in diagnostic imaging to help target radiopharmaceutical agents.^75^ THPs represent a step forward in cancer diagnosis and treatment.

### 3.2 Cell-Penetrating Peptides

CPPs are considered the least selective ligands for RME and are believed to translocate across cell membranes via a receptor-independent mechanism.^76^ Very recently, disclosures in cell-surface receptors responsible for cellular uptake of CPPs paved the way for the optimization and exploitation of CPPs as ligands.^77^

**Figure 7:**
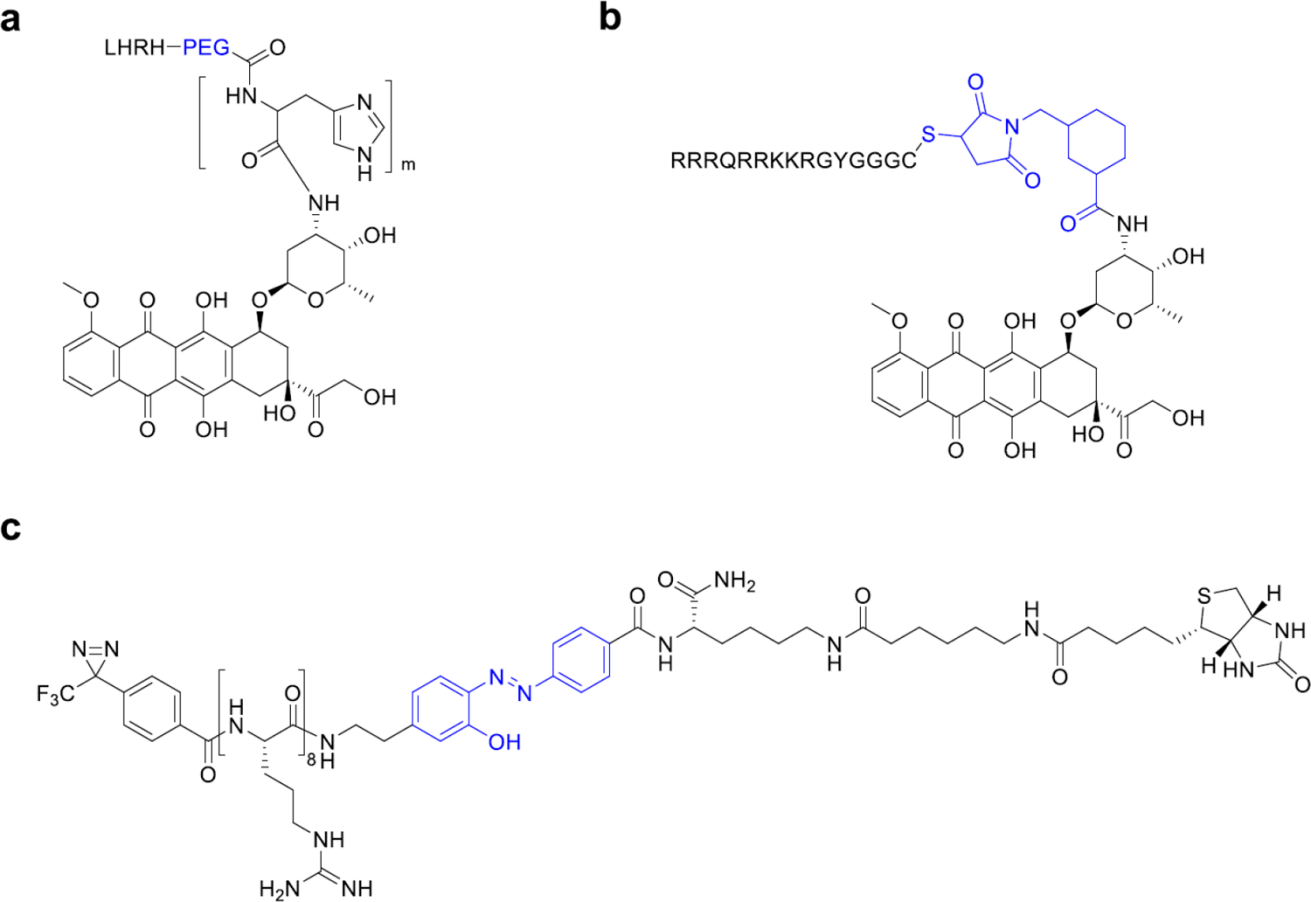
(a) LHRH-PEG-PHIS-**1**. (b) **1**-TAT conjugate. (c) Cleavable probe of octa-arginine peptide. Linker is colored in blue.

CPPs are cationic and/or amphipathic peptides of typically 8–30 amino acids and have been widely used to induce cellular uptake of bioactive cargoes.^78−80^ CPPs can be either covalently or noncovalently be coupled with a cargo. Identification of key amino acids to induce cellular uptake has been a pivotal parameter for the development of efficient ligands. The isolation of the active transporting peptide sequence within the HIV-TAT (TAT48–57: GRKKRRQRRR) represented a breakthrough for CPPs development. This sequence is called TAT peptide or TAT.^81^ Due to their high efficiency in internalization, arginine-rich CPPs such as oligoarginine and TAT facilitate the intracellular delivery of a wide range of cargoes, including peptides, antibodies, nucleic acids, nanoparticles, and small molecule drugs.^82^ Different studies have reported the pivotal role of arginine as a basic amino acid in CPPs, since it interacts with the guanidinium and phosphate groups at the cellular membrane. Indeed, the surface of cancer cells is known to be more negative with respect to that of normal cells. The negative charge generated on cancer cells is related to the different sugar metabolism pathways from normal cells due to the higher amount of lactic acid production.^83^ Positively charged CPPs bind through electrostatic interactions to the outside of cancer cells and promote RME.^84^ However, the widespread use of CPPs is hampered by the lack of specific selectivity. TAT has been shown to strongly enhance the intracellular delivery of **1**. Due to the nonspecific cell penetrating features of TAT, CPPs have been coupled to nanocarriers. Recently, Yang et al. developed acid-sensitive micelles as delivery method for TAT protection. The luteinizing hormone modified poly(ethylene glycol)-poly(l-histidine)-**1** (LHRH-PEG-PHIS-**1**, Figure 7a) micelles were employed to deliver **1**-TAT (Figure 7b). This strategy represents a step forward in the safer use of cytotoxic agents since the micelles dissociate in response to the tumor extracellular pH. Afterward, **1**-TAT can cross the cell membrane of tumor cells and elicit a cytotoxic effect.^85^

In 2018, an anionic cell-penetrating tetrapeptide, Glu-Thr-Trp-Trp (ETWW), with excellent potential for cell penetration, has been reported. The tetrapeptide has been coupled to liposomes to efficiently deliver **1** to the nucleus of cancer cells.^86^ Very recently, Dominguez-Berrocal et al. developed a chimeric trifunctional peptide composed of a CPP, a nuclear localization sequence, and a peptide blocking the interaction of the primary downstream effectors of the Hippo signaling pathway (TEAD and YAP). The novel peptide delivered the cargo specifically to the nucleus and showed an apoptotic effect in tumor cell lines. The antitumor efficacy in a breast cancer xenograft model is encouraging for the development of nuclear anticancer drugs.^87^

In addition to cancer treatment, nanoparticle-forming CPPs have been investigated in gene therapy approaches. However, CPP-mediated plasmid DNA (pDNA) delivery has been inefficient mostly because CPPs condense pDNA into nanoparticles that easily disintegrate, without delivering the therapeutic amount of pDNA into cells. In addition, CPPs and their cargo could be trapped into endocytic vesicles, preventing the pDNA from reaching the nucleus. These limitations can be overcome with the addition of a hydrophobic stearic acid residue since hydrophobic interactions are essential to form and stabilize the CPP/pDNA nanoparticles. Veiman et al. proposed pepFect14 (stearyl-AGYLLGKLLOOLAAAALOOLL, PF14, where O is ornithine) as a suitable non-natural peptide to form stable nanoparticles with pDNA. These nanoparticles could lead to an efficient gene delivery allowing the optimal transfection of genetic material into cells.^88^ The uptake of PF14 and other CPP/oligonucleotide (siRNA or pDNA) complexes is mediated by scavenger receptors (SCARA).^89^ These receptors bind promiscuously all negatively charged macromolecules and mediate their uptake.^90^

Another noteworthy application of CPPs is the delivery of neuroprotective peptides to the central nervous system for the treatment of neurological disorders. Arginine-rich CPPs show promising results in the delivery of neuroprotective peptides, especially to aid in treating cerebral ischemia and stroke. Several groups have shown that TAT and other arginine-rich cell penetrating peptides have intrinsic neuroprotective properties.^91,92^ Meloni et al. suggested that the neuroprotection might be related to carrier peptide endocytosis: neuronal cell surface structures such as ion channels and transporters are internalized during endocytosis, decreasing the calcium influx associated with excitotoxicity.^93^ In addition, endocytosis causes internalization of cell surface receptors leading to a decrease in receptor-mediated neurodamaging signaling pathways.^94^

Endocytosis has a crucial role in the cellular uptake of CPPs. Macropinocytosis^95^ and other classes of endocytosis such as clathrin-mediated^96^ and caveolae-mediated endocytosis^97^ are involved. Moreover, direct penetration of CPPs through plasma membranes has been described.^98^ Originally it was believed that CPPs translocated across cell membranes via a receptor-independent mechanism, leading to a not-cell-type-specific uptake.^76^ Very recently, Kawaguchi et al. identified syndecan-4 as a cell-surface receptor responsible for cellular uptake of octa-arginine (R8) peptide via clathrin-mediated endocytosis. A cleavable probe of the R8 peptide (Figure 7c) was used to identify syndecan-4 as an endogenous membrane-associated receptor.^77^ Even though this cell-surface receptor is ubiquitously expressed, it is overexpressed in breast and testicular cancer cells^99,100^ and in kidney cells of patients with IgA nephropathy.^101^

Rodriguez Plaza et al. proposed that CPPs work as cationic antibacterial peptides (CAPs) in the presence of bacterial cells. While CPPs enter eukaryotic cells without apparent toxicity, CAPs are able to make pores in the membrane and kill bacteria. Iztli peptide 1 (IP-1), showing both CPP and CAP activities, was utilized to explain this different behavior. IP-1, a hunter–killer peptide against *Saccharomyces cerevisiae*, makes pores only in the presence of high electric potential value at the membrane, which have been found in bacteria and mitochondria.^102^ Therefore, CPPs are able to switch from penetrating mammalian cells with any apparent toxicity to killing bacterial cells in the presence of large membrane potential.^102,103^

### 3.3 Tumor Homing Peptides

**Figure 8:**
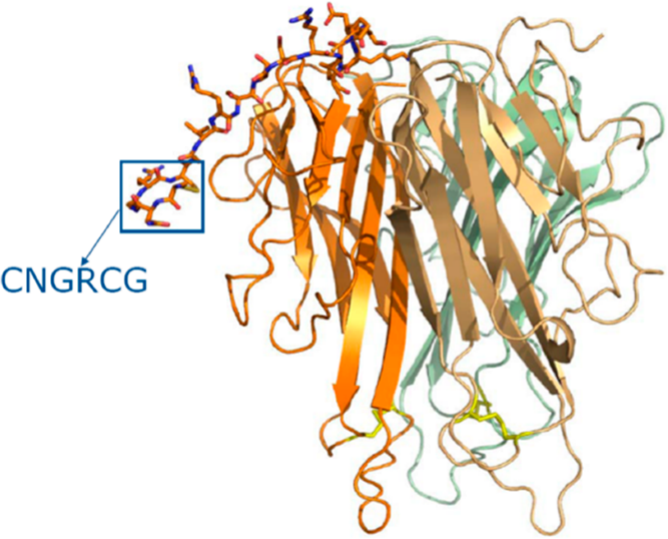
Structure of a monomer NGR-hTNF [https://www.molmed.com/node/33].

As described in section 3.2, the majority of CPPs lack specificity leading to reduced therapeutic efficiency and side effects. To overcome the limitations of CPPs, more specific peptides, namely, tumor homing peptides (THPs), have been developed.^104^ THPs are short peptides constituted by a few amino acids (3–15) and are considered a type of CPP. They have the intrinsic property to recognize oncological-specific proteins and molecular markers overexpressed on tumor cells or tumor vasculature.^105^ After binding to cell surface receptors, tumor homing peptides induce RME. Classical vascular-homing peptides are peptides containing the NGR motif, which binds to aminopeptidase N (CD13) or the RGD motif, which binds to α_ν_ integrins.^106^ Aminopeptidase N is overexpressed by endothelial cells of tumor vasculature and has been demonstrated to be involved in angiogenesis and cancer progression. Likewise, α_ν_ integrins are overexpressed in blood vessels in the tumor and represent a potential target to deliver cytokines to tumor vasculature. **1** was the first anticancer drug to be coupled to a NGR peptide. Later, phase I and phase II clinical trials of NGR-hTNFα as a single antitumor agent and in combination with **1** have been performed for a variety of cancers, including ovarian, colorectal, and small cell lung cancer (SCLC).^107,108^ Tumor necrosis factor α (TNFα) has demonstrated powerful antitumor activity but also severe toxicity. Conjugation of hTNFα with the tumor homing peptide NGR (Figure 8) improved safety and efficacy of TNF. Moreover, a synergism between NGR-hTNFα and chemotherapy was observed, since NGR-hTNFα has been shown to increase the intratumoral chemotherapy penetration.^73^ Very recently, phase II clinical results have been disclosed and NGR-hTNF plus **1** demonstrated promising activity in patients with relapsed SCLC.^74^ A phase III clinical trial was performed in patients with malignant pleural mesothelioma to assess the efficacy and safety of NGR-hTNF plus best investigator choice [NCT01098266]. Despite the positive results in phase II evaluation, the phase III clinical trial did not meet its end point; no significant differences in overall survival were observed between treated groups. However, further investigation is needed due to the poor prognosis of patients after first-line treatment.^109^

In addition to the TNF protein, the TNF gene has been employed for cancer gene therapy and has been reported to promote antitumor responses both in animal models and in patients. The plasmid DNA encoding CNGRCG-TNF and ACDCRGDCFCG-TNF (pNGR-TNF and pRGD-TNF, respectively) displayed growth inhibition of subcutaneous murine B16F1 melanomas and RMA-T lymphomas after intramuscular injection.^110^ RGD-TNFα was also evaluated for its ability to enhance the antitumor effect of chemotherapy; however NGR-hTNFα was mostly chosen for clinical trials. RGD has been preferentially exploited for diagnostic applications and many RGD-based radiopharmaceutical agents have been assessed for cancer imaging.^75^ Bispecific NGR peptides (GNGRAHA), targeting both CD13 and αvβ3 integrin in the endothelium of solid tumors, have been developed. In 2018, Seidi et al. combined the NGR peptide, GNGRAHA, with a truncated form of coagulase (tCoa) generating a bifunctional protein (tCoa-NGR) with novel anticancer properties. This strategy allowed selective targeting of the tumor neovasculature and inducing of selective thrombosis in tumor-feeding vessels. In tumor models, tCoa-NGR led to a significant reduction of tumor growth after systemic administration.^111^ Therefore, tCoa-NGR represents a promising anticancer strategy to induce tumor infarction and reduce systemic side effects.

Besides TNF, tumor homing peptides could facilitate distribution of other cytokines into tumor cells and enhance their therapeutic effect. In 2017, it has been shown that RGD enhances the antitumor effect of IL-24. Melanoma differentiation-associated gene-7/interleukin-24 gene (MDA-7/IL-24) is a unique tumor suppressor gene, which promotes selective apoptosis of cancer cells. RGD-coupled IL-24 construct induced apoptosis in hepatocellular carcinoma-related cell line.^112^ The results highlight the benefit of cytokine targeting by THPs to cancer cells. Coupling RGD to the N-terminus of IL-24 led to a stronger interaction with the receptors. On the contrary, adding RGD to the C-terminus of IL-24 disrupted native interactions and reduced the apoptosis induction properties.^113^ Very recently, Bina et al. confirmed these results with *in silico* targeting of RGD/NGR-modified IL-24 to tumor cells.^114^

THPs have shown potential to be versatile platforms of polymers for nonviral gene delivery.^115^ pDNA complexes of recombinant proteins with poly(l-lysine) and THP showed significant improvement of target specificity to cancer cells by additions of F3 and CGKRK THPs. F3 peptides are high affinity ligands for nucleolin, which is expressed on the surface of cancer angiogenic endothelial cells, and selectively bind MDA-MB-435 cells.^116,117^ CGKRK peptides were described to bind to heparin sulfate in cancer vessels.^118,119^

THP–gold nanoconjugates actively targeted MCF-7 cells in comparison to nontumor 3T3-L1 fibroblast cells.^120^ THPs specific for MCF-7 cells were selected from a phage display library, synthesized, and conjugated to spherical gold nanoparticles by a heterobifunctional cross-linker with an ethylene oxide spacer. This work proved the possibility of developing nanomaterials that can rely on tumor targeting potential irrespective of a specific knowledge of the target cell biology.

### 3.4 Monoclonal Antibodies

Over the past decade, monoclonal antibodies (mAbs) have significantly improved the clinical outcomes for cancer patients since they specifically bind tumor-associated target antigens and eventually deliver cytotoxic agents to tumor cells in a targeted manner while sparing normal cells.^121^ mAbs are conjugated to small-molecule chemotherapeutics, and the resulting antibody–drug conjugate (ADC) is parentally administered (intravenous or subcutaneous). After binding to their target antigens, ADCs are internalized through RME.^122^ The development of a procedure to produce mAbs has increased the enthusiasm of scientists for the development of precise targeted cancer therapy. Humanized and fully human antibodies have the advantage of being retained longer in circulation than their murine equivalents and led to a dramatic increase in the use of antibody-based drugs against cancer.^123,124^ However, many challenges have to be overcome for the development of optimized and functional antibody–drug conjugates with possible application as therapeutic agents.

One of the major challenges in the development of ADCs is to incorporate a linker able to preserve the ADC stability in systemic circulation for an extended period and to release the payload at the targeted site. Conjugation site and linker choice are key parameters in the pharmacokinetic properties of ADCs. The site of attachment to an antibody can also be engineered in different ways to incorporate a linker and subsequently a bioactive molecule.

**Figure 9:**
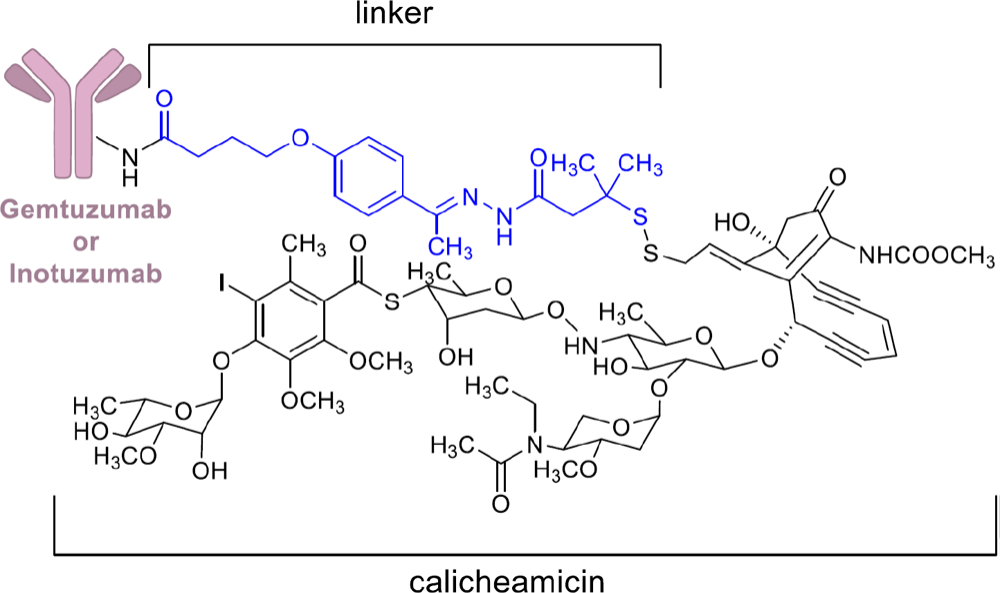
Chemical structure of **3** and **4**. Linker is colored in blue.

**Figure 10:**
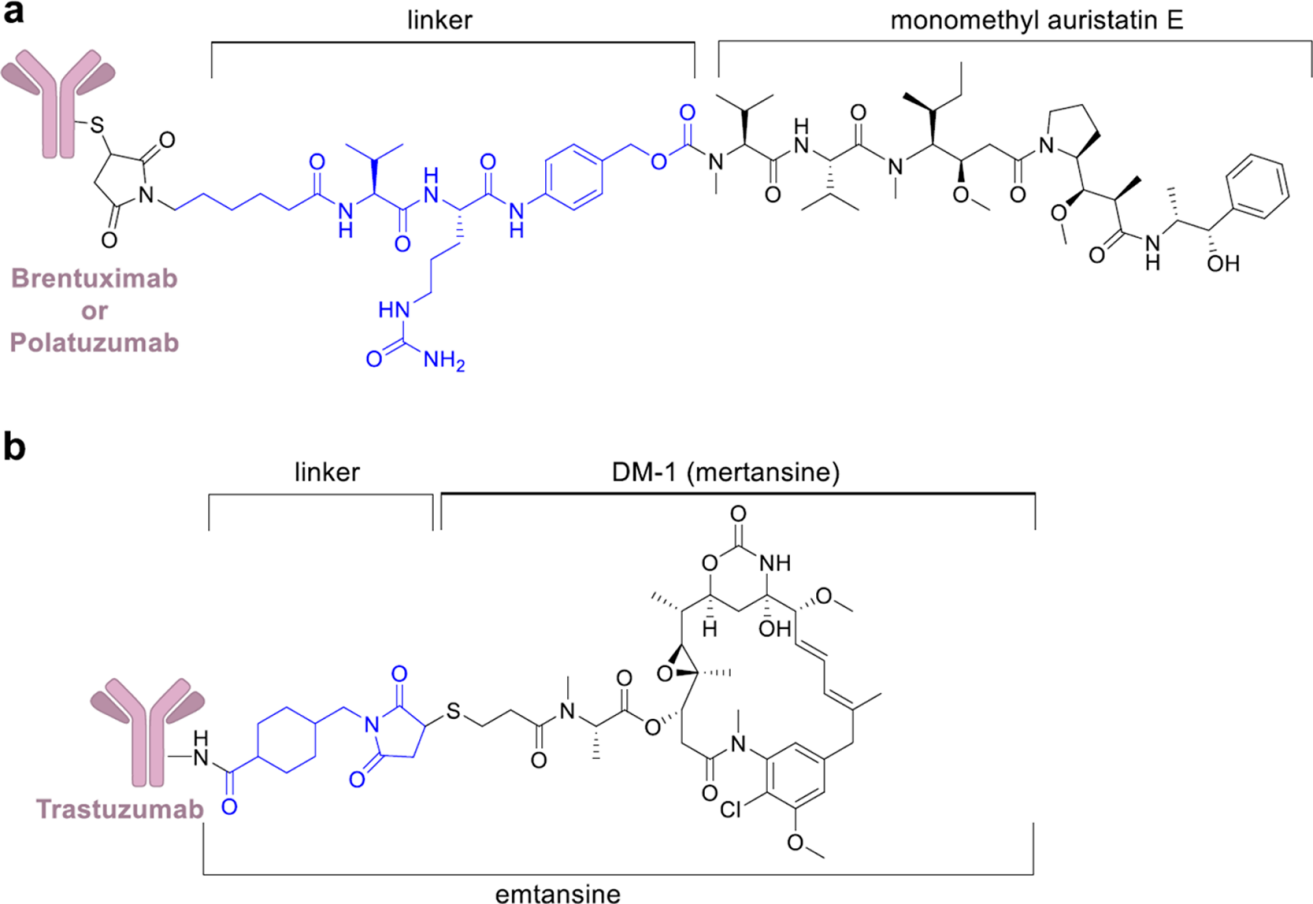
(a) Chemical structure of **5** and **7**. (b) Chemical structure of **6**. Linkers are colored in blue.

Considering the five ADCs approved so far by FDA, gemtuzumab ozogamicin (**3**) and inotuzumab ozogamicin (**4**) have an acid-sensitive hydrazone linker (Figure 9), brentuximab vedotin (**5**) and polatuzumab vedotin (**7**) have a lysosomal protease-sensitive peptide linker (Figure 10a), and trastuzumab emtansine (**6**) exploits a noncleavable SMCC (*N*-succinimidyl-4-(maleimidomethyl)cyclohexane-1-carboxylate) linker (Figure 10b).

Compound **3** uses side chain reactive lysines of a humanized anti-CD33 mAb to attach calicheamicin, a highly cytotoxic agent that induces double-strand DNA cleavage, by a bifunctional acid sensitive hydrazone linker (Figure 9). After being launched in 2000 as therapeutic agent for relapsed acute myelogenous leukemia, this ADC was withdrawn from the market due to the limited benefit over conventional anticancer treatment and the serious hepatotoxicity.^125,126^ This withdrawal increased the concern on the stability of the hydrazone linker. In addition, the ADC heterogeneous nature of the drug conjugate concurred to premature release of the conjugated payload, leading to a significant toxicity compared to traditional chemotherapy. Subsequent trials using a lower dose led, in September 2017, to the FDA approval of **3** for newly diagnosed and relapsed/refractory acute myeloid leukemia.^65,66^

Compound **4** is another antibody–drug conjugate of calicheamicin.^127^ It is formed by a CD22-directed monoclonal antibody covalently bonded to *N*-acetyl-γ-calicheamicin (Figure 9). **4** received FDA approval in 2017 to treat relapsed or refractory CD22-positive B-cell precursor acute lymphoblastic leukemia.^67^**4** has shown excellent activity in the clinic, and ongoing trials are evaluating its value as frontline treatment.^128^ A phase III clinical trials is assessing the benefits of treating newly diagnosed B-cell acute lymphoblastic leukemia with **4** in combination with chemotherapy [NCT03150693].

In 2011, compound **5** received approval for Hodgkin’s lymphoma (HL) and anaplastic large-cell lymphoma (ALCL).^68,69^**5** utilizes side chain cysteines to conjugate monomethyl auristatin E (MMAE), a potent antimitotic agent, with the anti-CD30 mAb (cAC10) through an enzymatically cleavable dipeptide (valine–citrulline) linker (Figure 10a).^129^ A selective reduction of the disulfide bonds in the four interchain provides up to eight reactive sulfhydryl groups that facilitate drug conjugation (drug to antibody ratios are from 0 to 8).^130,131^ Exploiting this method to link the drug, rather than using lysine conjugation, results in ADCs that could be easily purified and pharmacokinetically characterized. Besides its application in the treatment of different types of lymphomas, the safety and antitumor activity of **5** have been demonstrated also in patients with CD30-expressing solid tumors in a phase II clinical trial.^132^

In 2019, compound **7**, a second ADC of MMAE whose mAb targets CD79b (B-cell antigen receptor complex-associated protein β chain), was granted accelerated FDA approval for the treatment of adults with relapsed or refractory diffuse large B-cell lymphoma (DLBCL) in combination with bendamustine plus rituximab (BR).^72^ A multicenter phase Ib/II clinical trial including a cohort of 80 patients with relapsed or refractory DLBCL [NCT02257567] granted drug approval. At the end of the therapy, the complete response rate was 40% with **7** plus BR, compared with 18% with BR alone.^133^

Compound **6** uses a noncleavable SMCC linker to cross-link the warhead cytotoxic agent emtansine (DM1), a microtubule inhibitor, to lysine residues of anti-HER2 mAb, trastuzumab (Figure 10b). Lysine-MCC-DM1 complex, an intercellular metabolite, turned out to be as active as the parent drug, after trastuzumab degradation by lysosomes. It is clinically employed in patients with HER2-positive metastatic breast cancer.^70,71^ The approval of **6** for the treatment of breast cancer highlighted the capability of ADCs to target solid malignancies in addition to hematologic tumors. With the recent approval of **7**, there has been a boost in research investigating the use of ADCs in cancer treatment. ADCs are likely to become a pivotal part of future targeted cancer therapy.

**Figure 11:**
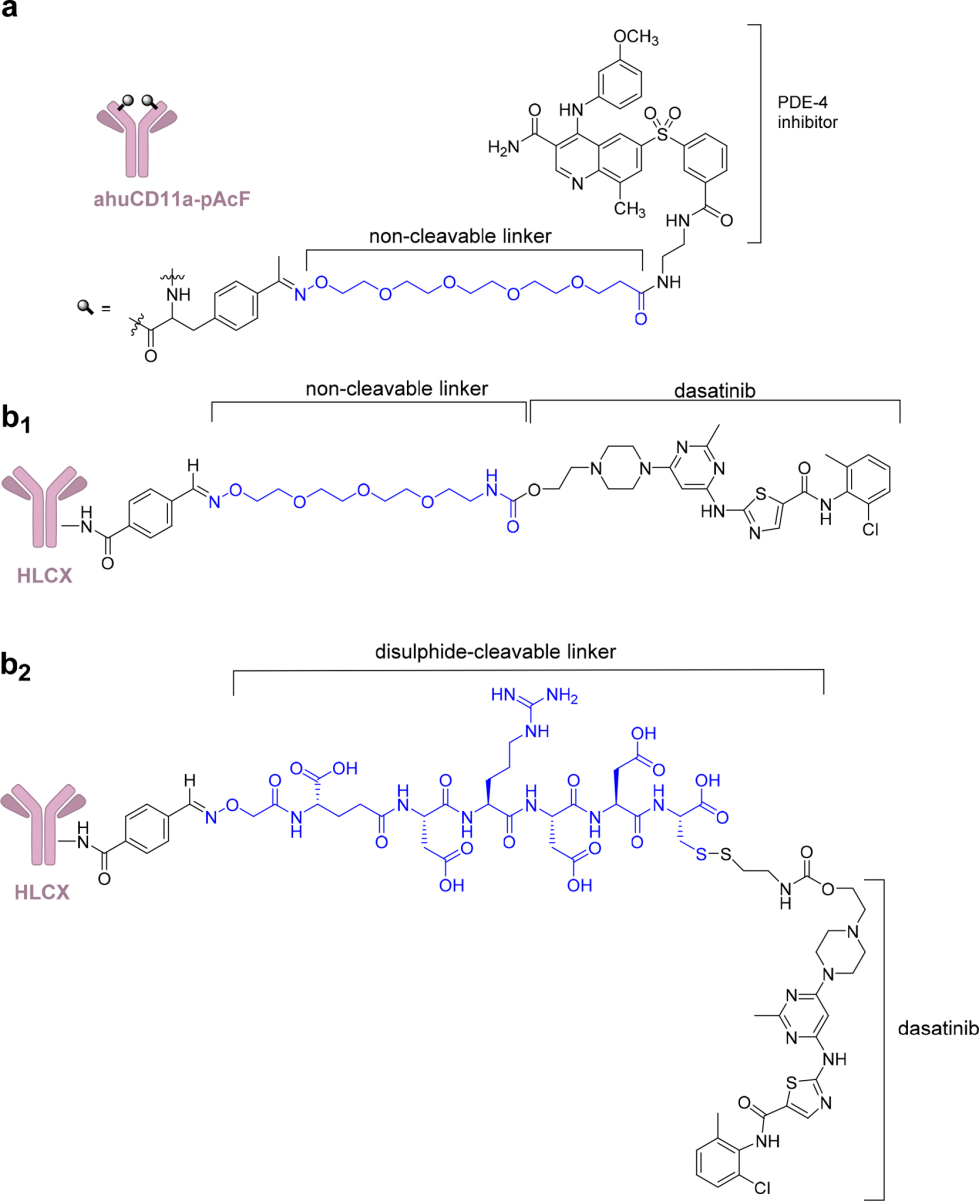
(a) Anti-inflammatory human αCD11a antibody conjugated to a PDE4 inhibitor. (b_1_, b_2_) HLCX, immunosuppressive humanized antibody that binds selectively to CXCR4, conjugated to **8** with a noncleavable linker (b_1_) and a disulfide-cleavable linker (b_2_). Linkers are colored in blue.

Although a huge effort has been made to produce ADCs for oncology, they are also an attractive platform to deliver noncytotoxic bioactive cargos in a cell-specific way aiming to reduce potential side effects related to off-target interactions. For example, an antibody–drug conjugate that selectively recognizes immune cells through the CD11a antigen has been conjugated to a derivative of a highly potent phosphodiesterase 4 (PDE4) inhibitor (GSK256066) (Figure 11a). This strategy could limit neurological side and gastrointestinal toxicity that have hampered a broad application of PDE4 inhibitors.^134^ To obtain a site-specific conjugation to the anti-human CD11a antibody, the unnatural residue *p*-acetylphenylalanine (pAcF) was linked to the heavy chain of efalizumab (site A122). To enable conjugation of GSK256066, a linker containing a tetraethylene glycol spacer with a terminal aminooxy group was reacted under slightly acidic conditions with the pAcF ketone, resulting in stable covalent conjugates. Conjugation was performed with drug/antibody ratio of 2 (1 bioactive molecule linked to each heavy chain). Recent studies have supported the feasibility to develop mAbs-PDE4 inhibitor conjugates as promising therapeutics for treating ulcerative colitis due to the specific delivery of immune suppressants to immune compartment.^135^ In addition, autoimmune diseases represent a potential field for ADCs application and significant advancements have been done in the past decade. Wang et al. proposed the use of dasatinib (**8**), a Bcr-Ab1 tyrosine kinase inhibitor, for immune suppression and developed an immunosuppressive ADC (Figure 11b) which targets CXCR4 and delivers **8** to human T lymphocytes. Modeling and structure–activity relationship studies highlighted that the hydroxyl moiety of **8** is not required to observed pharmacological activity.^89,90^ Therefore, it was modified for conjugation to the antibody with a noncleavable linker by reaction with *p*-nitrophenyl chloroformate and carbamylation with a tetrapolyethylene glycol (PEG) linker displaying an aminooxy group. The resulting **8**–antibody conjugate inhibits T cell receptor (TCR)-mediated T cell activation and cytokine expression with nanomolar EC_50_ and shows minimal effects on cell viability. This strategy could lead to an improved efficacy and safety of kinase inhibitors and to their exploitation in nononcological diseases.^136^ A phase II clinical trial is currently ongoing to determine the benefit of **5** in the treatment of systemic sclerosis, a multisystem autoimmune disease [NCT03198689].

These results highlight that, besides cancer, ADCs have potential application in a wide range of inflammatory and autoimmune disorders. Naked therapeutic antibodies have launched a novel era of both autoimmune disease and cancer treatment, but ADCs represent the next-generation antibody therapies and will represent a breakthrough in the treatment of these illnesses.

The ability of monoclonal antibodies to selectively bind tumor-associated target antigens and release cytotoxic agents to the tumors in a targeted manner has dramatically improved the clinical practice. Further advancements in this field will lead to the success of precise targeted cancer therapy. RME is also exploited by CPPs and THPs, but they have a poor selectivity compared to antibodies. However, nanocarriers are attractive tools to be coupled to CPPs and THPs and improve their safety and selectivity. In addition, the use of nanocarrier is boosting the antibody-based delivery of biological cargos into cancer cells. In the future, these systems are expected to become essential therapeutics for the treatment of malignancies and central nervous system disorders. Moreover, THPs are essential components of radiopharmaceutical agents and will represent a step forward in cancer diagnosis.

## 4 Chimeric Compounds and Target Engagement

### 4.1 Target Engagement

One of the main failures in translating preclinical results into a positive clinical outcome is the lack of pharmacokinetic and pharmacodynamic validation of drug–target interactions *in vivo* with serious impact on efficiency and costs of the drug discovery process. The mechanism that small molecules adopt to engage their targets inside living cells is a crucial step in medicinal chemistry and chemical biology, since it requires the availability of appropriate assays and inhibitors/ligands. The molecular recognition event in living cells between drugs and targets is defined as “target engagement”, and the associated technologies represent a rapidly evolving field of research.^137−140^

This technology allows target validation in living systems: in cells, tissues, and animal models. It confirms compounds cellular entry and target binding and can suggest optimized drug delivery to enable compounds to be more effective, specific, bioavailable, and less toxic.^2,141,142^*In vitro* studies could foresee the optimization of human performance characteristics.^143^ To perform these studies, selected leads and drugs should be conjugated with appropriate tags to obtain chimeric compounds with a diversity of structures (see Figure 1).^2,141,142^

To perform a target engagement study, it is essential to (i) know the target localization into the cell, (ii) design an assay for cellular setting, (iii) ensure the detection of the observable changes on cellular surface or intracellularly, depending on targets location, and (iv) ensure the escape of off-targets and background noise of the cellular matrices.^144^ While quantification of compound binding to purified proteins or surface receptors (in particular to GPCR) is well established,^145,146^ the interaction of compounds with intracellular targets is difficult to quantify.

Regarding the fluorimetric detection, a fluorescent probe has to be covalently conjugated with the inhibitor (see section 2.4)^2,141,142^ and should show sufficient solubility (slightly different from the values required for a drug) and a log *P* of around 3, necessary for a suitable drug or inhibitor tagging. Lipophilicity may influence the amount of compound able to enter the cell and consequently available for binding. In addition, the fluorescent tag module should not mask the compound affinity for the target (see section 2). Target engagement assays might be invasive since they drive the intracellular environment away from equilibrium conditions.^147−149^ Orthogonal assays are usually needed to validate the results.^150^ Aktinson and co-workers studied the interaction of selective autophagy receptors with two conserved hydrophobic pockets (called W-site and L-site) of mATG8 (autophagy receptors to autophagy related 8) proteins through a linear residue, namely, the LC3-interacting region (LIR). Fourteen LIR-containing peptides were designed and synthesized, and their affinity for mATG8 was investigated using a competitive time-resolved FRET (TR-FRET). The assay used a GST-tagged mATG8 protein and a terbium labeled anti-GST antibody to measure the equilibrium dissociation constant, *K*_d_, by TR-FRET. The results were confirmed by additional structural information using nuclear magnetic resonance (NMR) spectroscopy. This work points out the importance of having two assays that exploit different experimental readouts to validate the results.^150^

A similar approach was reported to discover inhibitors of the signal-regulatory protein (SIRP)α-CD47 interaction with a high-throughput screening approach.^151^ CD47 is an immune checkpoint that downregulates the functionality of both innate and adaptive anticancer immune response through its SIRPα receptor. A series of small molecule ligands that selectively target SIRPα interactions with CD47 was discovered. The assay was performed using a specific LANCE TR-FRET assay and a ∼90 000-compound library. In parallel, an AlphaScreen based on similar TR-FRET technology was adopted for validation purposes. SIRPα was biotin tagged, and an antibody with the energy donor reagent was the tagged chimeric biomolecule exploited in the assays.

In the following subsections, target engagement technologies and examples of the use of tagged compounds are described.

### 4.2 Strategies Based on Small Molecule and Target Protein Modification

The proximity between a bioactive small molecule and its targeted protein can be studied using spectroscopic methods such as fluorescence or bioluminescence resonance energy transfer measurements (FRET and BRET, respectively). FRET and BRET occur only when the donor and acceptor are in close proximity (2–6 nm) and are unique methods to inspect intermolecular protein interactions and protein–ligand interactions in cells.^152^

**Figure 12:**
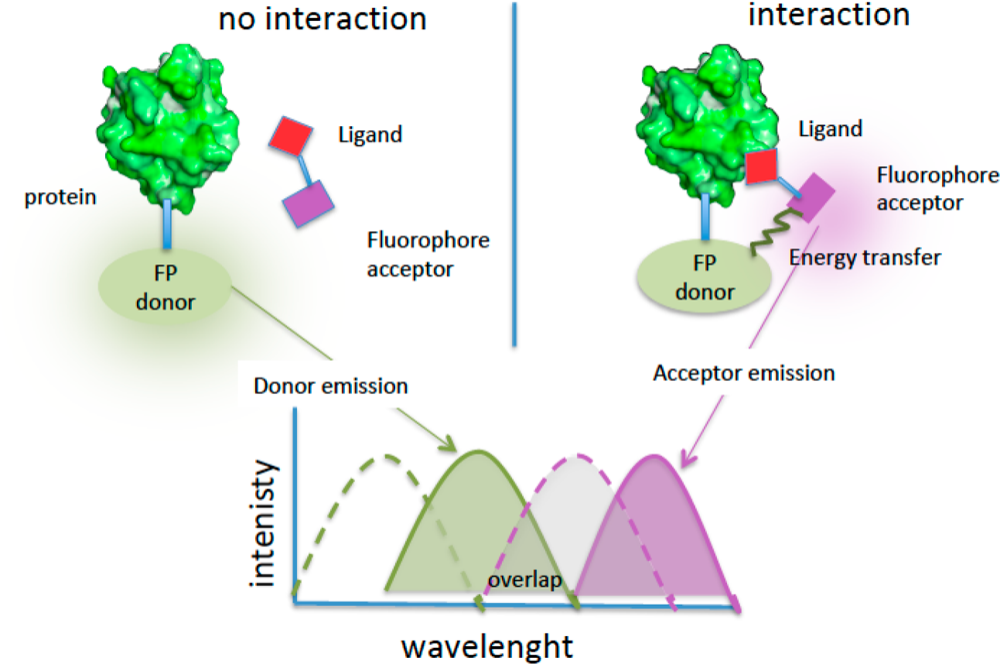
Description of FRET experiments for target engagement with no interaction (left) and interaction (right): FP, fluorescent protein; green dot line = donor excitation spectrum; green line = donor emission spectrum; violet dot line = acceptor excitation spectrum; violet line = acceptor emission spectrum.

In FRET (fluorescence resonance energy transfer or Förster resonance energy transfer) studies, a donor fluorophore upon excitation transfers energy to a nearby acceptor fluorophore. When a suitable acceptor is present, the donor emission is quenched and emission of light occurs at a longer wavelength (Figure 12). The essential criteria to observe FRET are (i) suitable distance, (ii) appropriate donor/acceptor orientation, and (iii) large overlap of the donor emission spectrum and the acceptor absorption spectrum. FRET can be quantified determining the change in donor fluorescence lifetime through fluorescence lifetime imaging microscopy (FLIM) based on FRET readout.^153−155^ FRET-FLIM monitors target engagement in living cells and provides details on the temporal and spatial distribution of the ligand–protein complex.

In some cases, a fluorescent protein (FP) is fused in cells to the target protein, and a FRET signal is generated when fluorophores are in close proximity (Figure 12, left). In a different protocol, the target protein can be ectopically expressed in the same cells and modified in a specific residue in order to bind a suitable fluorescent donor (or acceptor) (Figure 12, right).^156,157^ The target protein could be properly engineered to allow binding detection, for example, with a tetracysteine tag.^156,157^ Cells expressing the target protein coupled to a FP are treated with the a small molecule labeled with a fluorescent dye; subsequently the lifetime distribution of the donor fluorophore into a cell is determined. The donor fluorescence lifetime reveals the interaction sites into a cell as well as the areas with a reduced donor lifetime.

**Figure 13:**
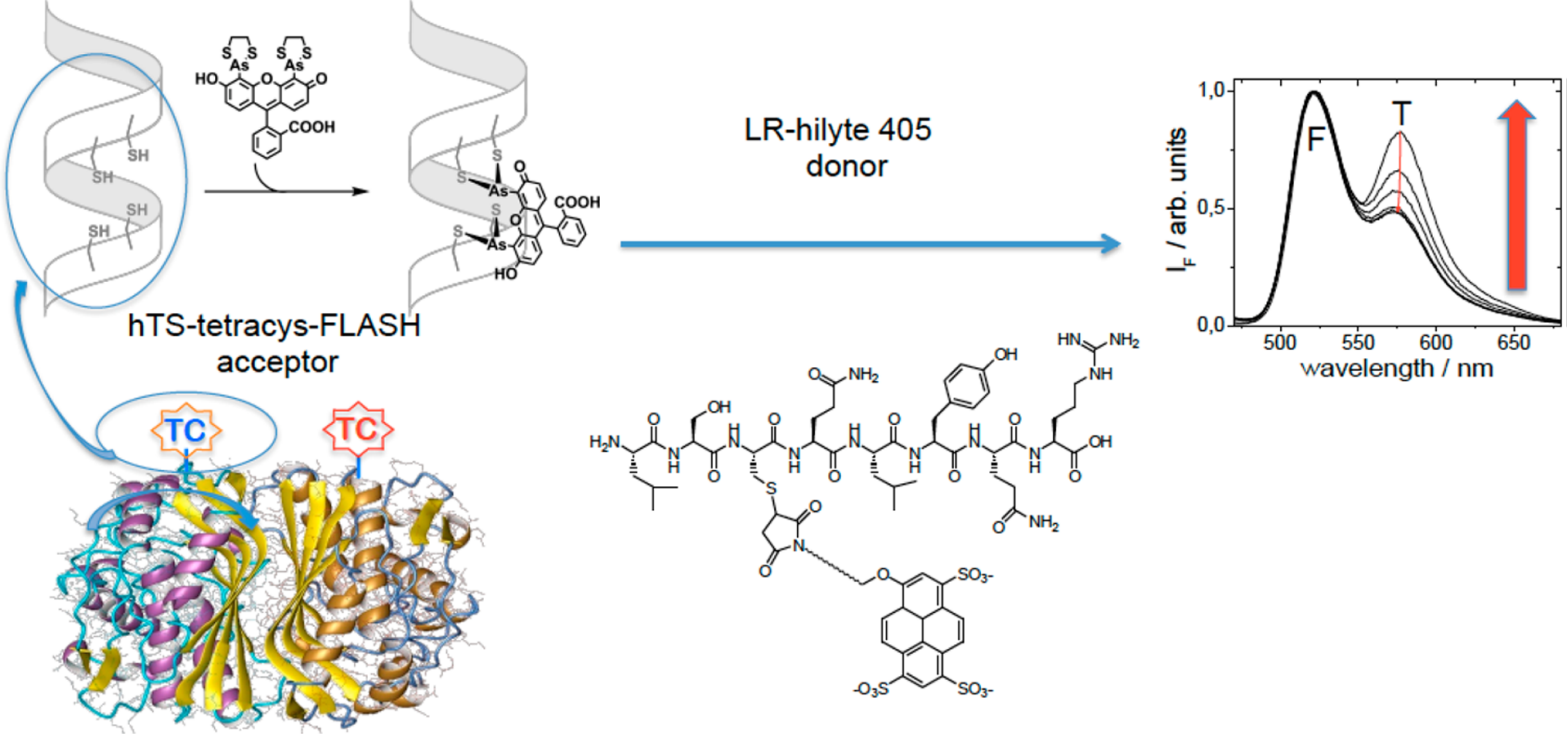
FRET experiment with TS dimer. The N-terminus is modified by inserting the sequence CCGPCC-tetracysteine (TC). Probe excitation at the proper wavelength causes the energy transfer and FRET signal increases upon binding of the LR-hilyte-405 ligand.

FRET based technology has been exploited, for example, for the recognition of phosphodiesterase^158^ and thymidylate synthase (TS) by tagged inhibitors. TS is an obligate homodimeric enzyme, and a tetracysteine (TC4) tag is introduced at the N-terminus. The fluorescein diarsenical probe FlAsH, added to the HEK-293 cell lysate containing the ectopically expressed protein, is coordinated by the tetracysteine behaving as a fluorescence donor. The tagged substrate is an octapeptide (LR) and is conjugated with the fluorescence acceptor probe Hylite-405 (Figure 13). Titration of hTS-tetracys-Flash (acceptor) with LR-hilyte 405 (donor) in lysates of cells transfected with hTS-tetracys shows an increase in FRET signal.

TR-FRET has been applied to the assessment of Bruton’s tyrosine kinase (BTK) occupancy in the clinical trials of tirabrutinib (**9**). Compound **9** (GS-4059/ONO-4059) is a second-generation, irreversible BTK inhibitor explored for the treatment of lymphoid malignancies. The inhibitor was conjugated with biotin through a carbamide–PEG mixed linker, and free and total BTK levels were measured using TR-FRET.^154^

### 4.3 BRET Experiments

**Figure 14:**
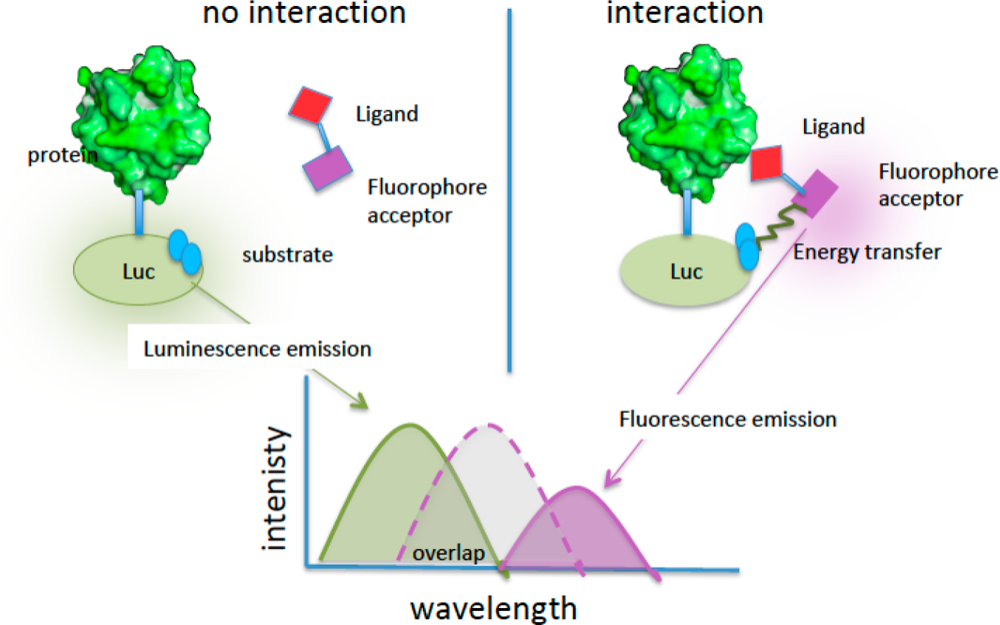
Description of BRET experiments for target engagement with no interaction (left) and interaction (right): Luc, luciferase; green line = donor emission spectrum; violet dot line = acceptor excitation spectrum; violet line = acceptor emission spectrum.

BRET (bioluminescence resonance energy transfer) is a mechanism describing the energy transfer between a donor (luciferase) and an acceptor (fluorescent) molecule. The spectral separation between donor and acceptor excitation required in FRET (Figure 13) is not required in BRET since the production of light originates from a chemical reaction catalyzed by the donor enzyme (Figure 14, right). Since BRET does not require the use of excitation illumination, it has advantages over FRET. BRET is therefore more applicable to the analysis of photoresponsive cells or cells that are easily damaged by excitation light. BRET has been exploited to detect protein–protein interactions in real time in living cells.^159,160^ In target engagement studies, cells express the target protein fused to a luciferase, while a ligand with a fluorophore tag behaves as an acceptor (Figure 14). Different BRET techniques are known and differ for the combination of the donor/acceptor/substrate used.^161^ Distance, orientation, and spectral overlap are the major parameters that influence both BRET and FRET. However, external excitation of the donor is not required in BRET; therefore phenomena related to simultaneous donor/acceptor excitation, fluorescence of the background, and photobleaching are not occurring. A microplate luminescence/fluorescence reader is one of the major components of the BRET imagining microscopy system, and the acceptor fluorescence is detected as readout. BRET allows determination of the affinity of a small molecule for the target protein and the study of the intracellular residence time of inhibitors using kinetic measurements. This method was exploited to prove the isoenzyme-specific engagement of histone deacetylase inhibitors^144^ and ligand engagement of G-protein-coupled receptors (β_2_-adrenergic and adenosine receptors).^145^ Robers et al. exploited a Nanoluc small luciferase protein (19 kDa) as a BRET donor instead of luciferase (Luc), since it showed a higher fluorescence yield, a narrow spectrum, and a stable luminescence. As a BRET acceptor, the non-chloro-TOM dye (NCT), showing membrane permeability and significant spectral resolution, was employed. To explore the interaction of intracellular engagement of HDAC inhibitors, the hydroxamate-based inhibitor (SAHA) was conjugated with NCT and was used as displacement substrate (tracer displacement by unlabeled compounds).^144^

The same authors reported the quantitative aspects relevant to fully characterize the engagement. Inside living cells, a NanoLuc-tagged intracellular protein of interest achieves a dynamic equilibrium with a cell-permeable fluorescent dye (tracer). After binding of an unlabeled small molecule, complex disruption leads to a loss of BRET signal that is detected in a microplate format (Figure 14). NanoBRET tracers are often produced starting from a drug or a tool compound and allow a quantitative measurement of the apparent affinity and a real-time assessment of the residence time.^162^

The BRET method was also adopted for the identification of antimicrobial hits targeting the protein–protein interaction between the initiation factor σ and the β′-subunit of bacterial RNA polymerase.^70^ The study combined an *in silico* screening with an *in vivo* bioluminescence resonance energy transfer in yeast cells, showing the large applicability of this technology. One hit was identified and optimized using medicinal chemistry approaches.^163^

**Figure 15:**
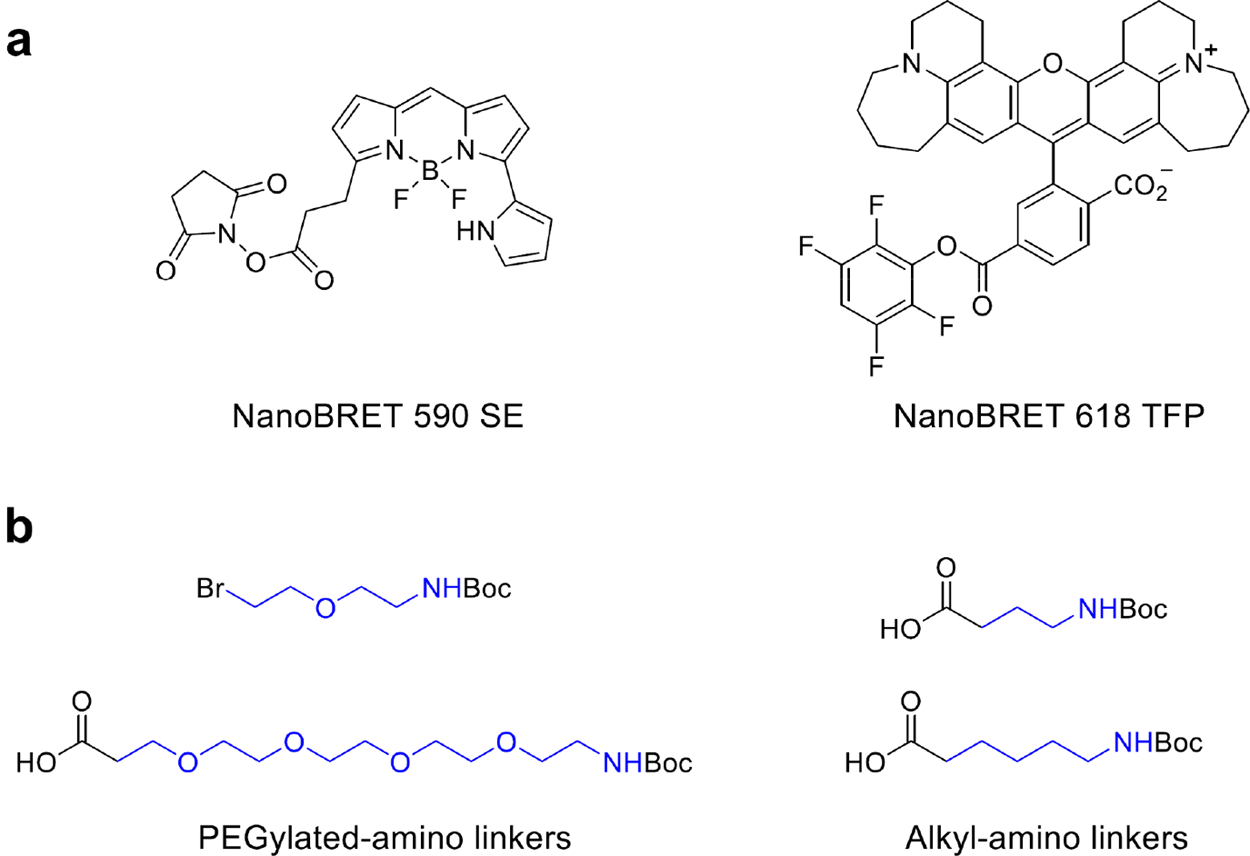
(a) Chemical structure of NanoBRET ester activated dyes; (b) example of linker building blocks. Dyes directly bound to the building block are known; however sometimes a linker (colored in blue) between the head tag and the fluorescent probe is necessary.

The description of the quantitative, real-time measurements of intracellular target engagement using energy transfer is reported, and NanoBRET tracers with optimized cell permeability have been developed and fully characterized (Figure 15a). Two main classes have been identified that represent robust chemical tools for the assay. Linkers between the dye and the reacting group such as succinimide (NanoBRET 590 SE) or reactive esters (nanoBRET 618 TFP) can influence tracer properties, including affinity and cell permeability.

### 4.4 A Chemical Proteomic Approach for Covalently Binding Ligands

**Figure 16:**
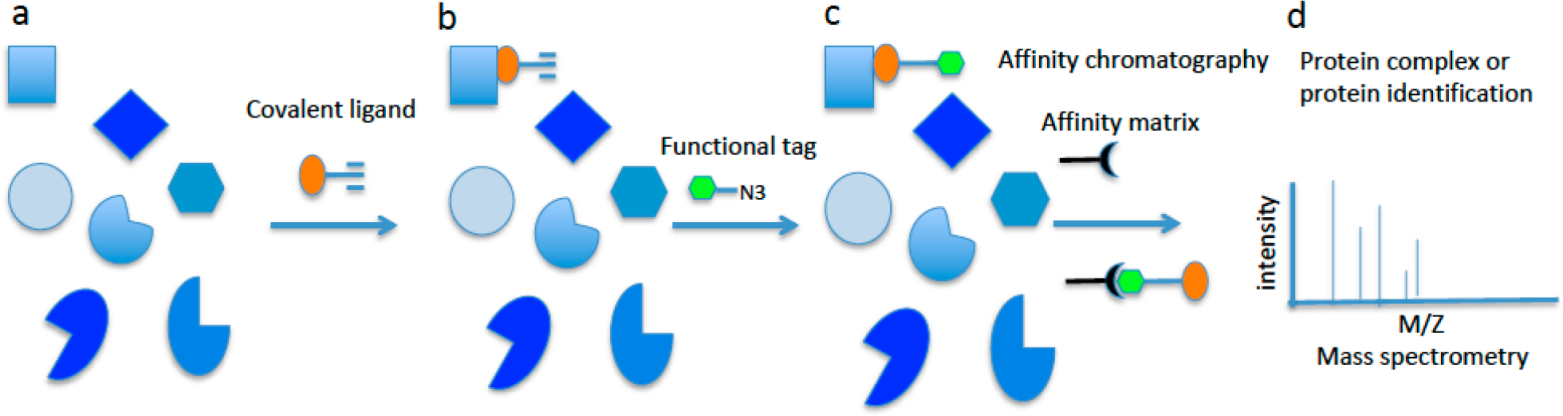
Example of ABCP process for covalently binding ligands. (a) Different targets available in the cells for binding the covalent ligand (orange) with the alkyne reactive functionality. (b) Covalent ligand binds the target and forms a covalent or noncovalent complex. The reactive group is exposed outside the binding site. (c) The functional tag (green) is added. It reacts with the reacting group of the covalent ligand (alkyne) with a click chemistry reaction, thus forming a covalent complex with the target. The green tag has a high affinity for the resin that should sequester the chimera–target complex from the sample matrix. (d) The affinity resin is added. The complex, once detached from the resin, is analyzed through mass spectrometry and the biomolecular target identified.

Affinity-based chemical proteomics (ABCP) is a method to study proteins or ligand–target interactions, based on protein isolation by an affinity reagent that can be coupled to a reporter system for detection. Affinity-based chemical proteomic has been used in target engagement studies of small-molecule drugs that covalently react with their targeted protein.^164,165^ The compounds have a chimeric nature since a reactive functionality such as an alkyne (Figure 16a) or azide group is introduced in a suitable position of the scaffold. After addition to the cell, the ligand reacts with the protein target bearing a reactive group exposed and regioselectively placed. Both wild type and mutant proteins can be exploited for the study. When the functional tag (or affinity tag) is added to the cell, it binds to the covalent ligand through a click chemistry reaction (alkyne with azide, Figure 16b). “Click chemistry”^20^ is exploited to attach *in situ* a functional tag, such as biotin. The functional tag allows affinity purification of the covalently bound protein of interest using, for example, streptavidin beads (Figure 16c), and protein identification is performed using tryptic digestion and nanoliquid chromatography–tandem MS analysis (Figure 16d). The functional tag presents a reactive head for the ligand and an affinity tag for the resin to allow affinity chromatography.

This method can be applied also to probes that bind proteins in a reversible fashion by the addition of a photoreactive group for UV detection of probe–protein interactions in cells (see photoaffinity labeling).^166^ ABCP allows also off-targets detection in cells.^167^

Wong et al. investigated the specificity for a series of ATP-competitive bivalent kinase inhibitors targeting ABL1.^168^ They proved the affinity and selectivity of bivalent inhibitors against Abl protein kinase with respect to other off-targets using dual functional chemical proteomics probes. A bivalent inhibitor A-2 showed high affinity together with improved selectivity over the parental ATP-competitive inhibitor.

**Figure 17:**
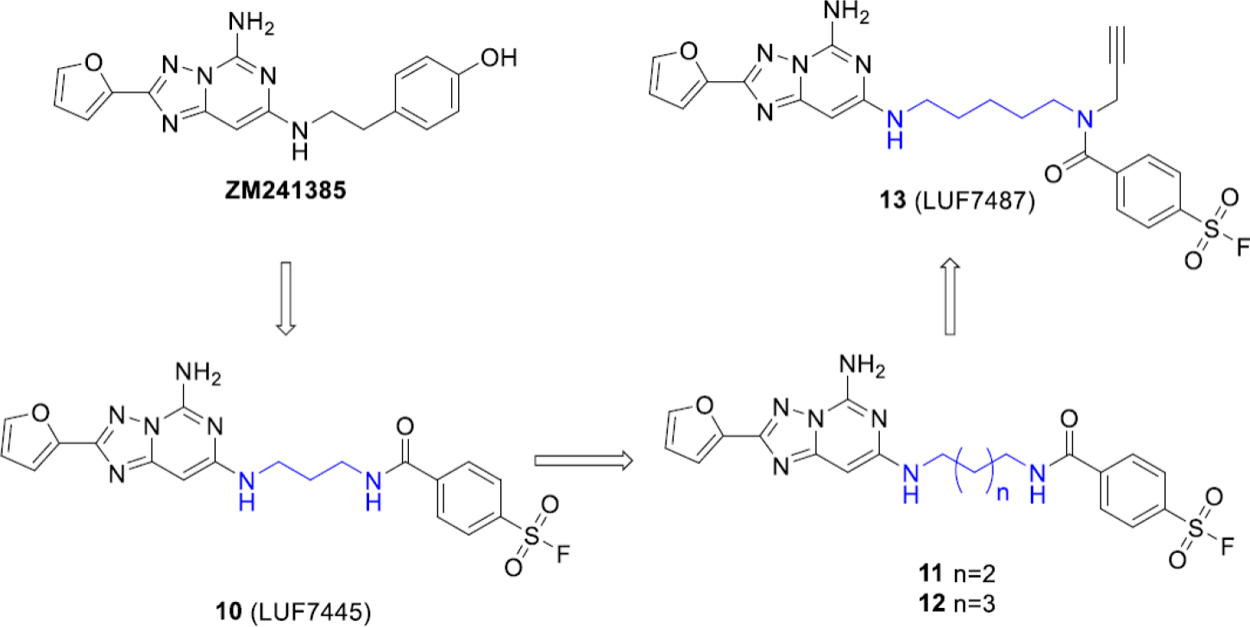
Chemical structures of the hA_2A_R antagonists investigated by Yang et al. The selective hA_2A_R antagonist (ZM241385) guided the design of the covalent antagonist **10**. The authors assessed the importance of the linker length (colored in blue) between the scaffold and the head on affinity and synthesized the optimized compounds **11** and **12**. Starting from compound **12**, the affinity-based probe **13** with an alkyne ligation-group and a fluorosulfonyl electrophilic was synthesized.

Another example of the pivotal role of chemical proteomic in chemical biology is the use of activity-based protein profiling (ABPP) to study proteins in their native environment.^169^ By exploitation of click chemistry, an affinity-based probe for the human adenosine A_2A_ receptor (hA_2A_R) was developed to investigate the structural biology of the G-protein-coupled receptor (GPCR). Yang et al. developed compound **10** (LUF7445), a clickable affinity-based probe, with an electrophilic reactive group, as a covalent antagonist of hA_2A_R. LUF7445 was discovered through chemical modification of compound ZM241385 introducing a fluorosulfonyl group, and different linker lengths have been investigated. On the most potent ligand, an alkyne-click handle was introduced leading to the synthesis of probe **13** (Figure 17). The binding of the ligand to the receptor was washout-resistant. This probe allowed assessment of the presence of hA_2A_R in complex biological samples. The identification of the affinity probe for a GPCR is a promising tool to monitor the endogenous GPCR expression related to human diseases.

### 4.5 Photoaffinity Labeling

**Figure 18:**
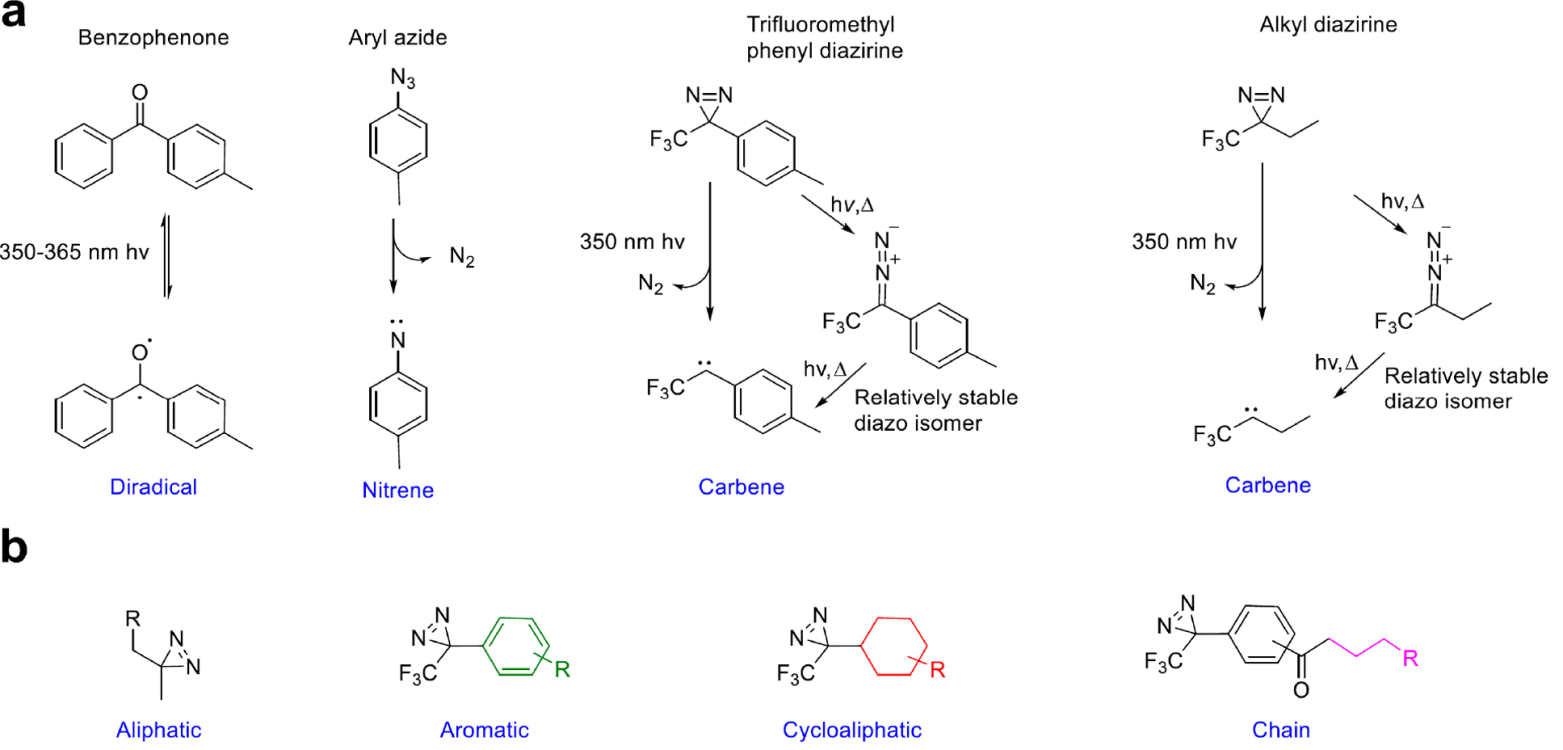
(a) Most relevant photoaffinity compounds used to tag protein ligands aiming to study target engagement, protein functions, and their photoreaction: benzofenone, aryl azide, and diazirine (trifluoromethylphenyl diazirine, trifluoromethylethyl diazirine). (b) General structures of scaffolds for photoaffinity tagging.

Photoaffinity labeling (PAL) is a well-known technique used to study specific protein function or inhibition.^170^ Photo-cross-linkers are conjugated with drugs or substrates that can bind to the target protein (protein of interest). Typically, photo-cross-linkers are (i) benzophenone (BP), (ii) aryl azide (AA), and (iii) diazirine (DA) (Figure 18). Upon photoirradiation, the photo-cross-linking functional group generates highly reactive species that react with adjacent molecules, leading to a direct covalent modification.^171^

PAL can capture partners through noncovalent interactions and explore the ligand accessible protein space in a selective mode. Photo-cross-linking agents have turned out to be essential tools to study difficult targets such as protein–protein interactions. Despite the high significance and extensive application, only few photo-cross-linkers are currently available. In the 1970s, BP has been introduced as a photo-cross-linker and is the most used in PAL due to the good selectivity and affinity toward methionine. Upon irradiation by 350–365 nm wavelengths, BP is converted into an active diradical. It reacts with protein functional groups exploiting an abstraction–recombination reaction mechanism. Aryl azides cross-link through nitrene, a reactive species, that is generated by loss of N_2_ upon photoirradiation with 254 and 400 nm wavelengths. Nitrene reacts with nearby C–H and heteroatom–H bonds, creating a novel covalent product. AAs are known to be chemically stable and to have superior photophysical properties than the corresponding acyl and alkyl analogs. Trifluoromethyl phenyl DAs and alkyl DAs can both produce carbene as reactive species losing N_2_ upon photoirradiation at 350 nm. They can form covalent adducts as phenyl diazirine (Figure 18a).^172^ Novel functionalized scaffold to be included in the chimeric compounds can be designed starting from different precursor reagents. In Figure 18b the colored fragments are included in the final chemical photoaffinity reagent.

**Figure 19:**
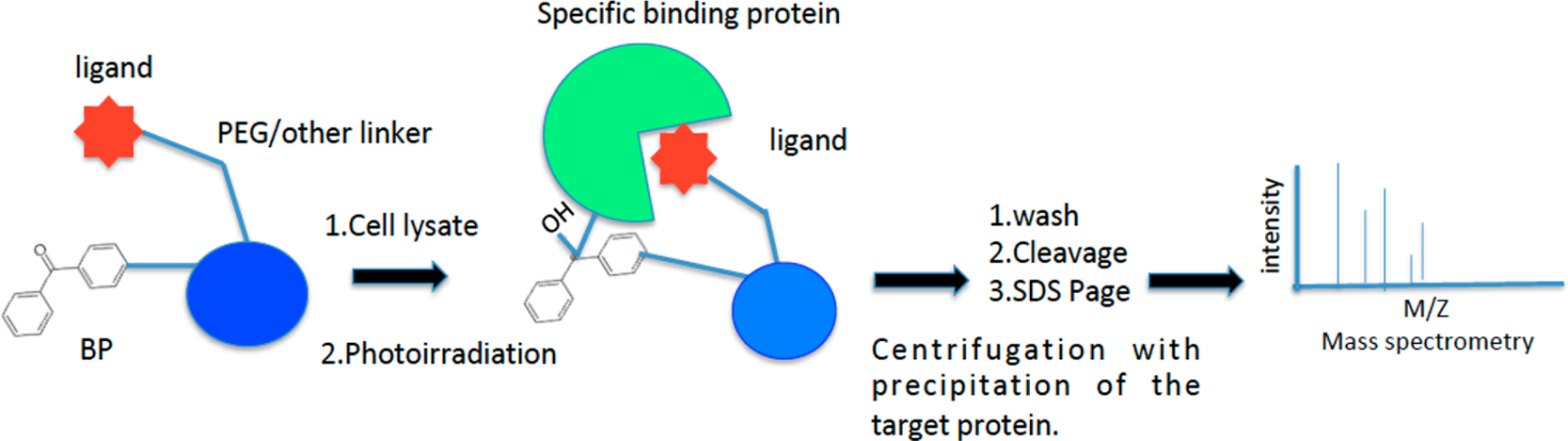
Efficient PAL method for protein identification using a bead-based multivalent probe.

A typical methodology for target deconvolution in drug discovery is applied to living cells or protein complexes, including cell lysates, that are incubated with the compound. The derivatized compound has a photoaffinity linker and a reacting agent (drug, inhibitor, or ligand), and the compound–protein binding is fixed by UV irradiation.^173^ Affinity tag is used to isolate proteins covalently bound to the compound, which are then analyzed exploiting MS-based proteomics for proteins identification (Figure 19). T. Tomohiro identified pyruvate carboxylase and C-terminal biotin carboxyl carrier protein as biotin-binding protein from HeLa cells using a PAL-based enrichment with an isotope-coded fluorescent and photocleavable tag followed by MS.^174^ Relatively small functional groups for click chemistry have been recently introduced aimed at improving the photo-cross-linking yield and at gaining sensitivity of MS-based proteomics. This method could be applied also when the affinity between the target protein and the small molecule is weak.

The Kaori Sakurai group used PAL to detect the binding protein of benzenesulfonamide.^175^ They produced trifunctional probes bearing a lysine scaffold and containing a benzenesulfonamide moiety as protein-binding ligand. The photoactivatable group was BP, and biotin was selected as reporter group, allowing the detection of the protein–covalent adducts (Figure 19).

**Figure 20:**
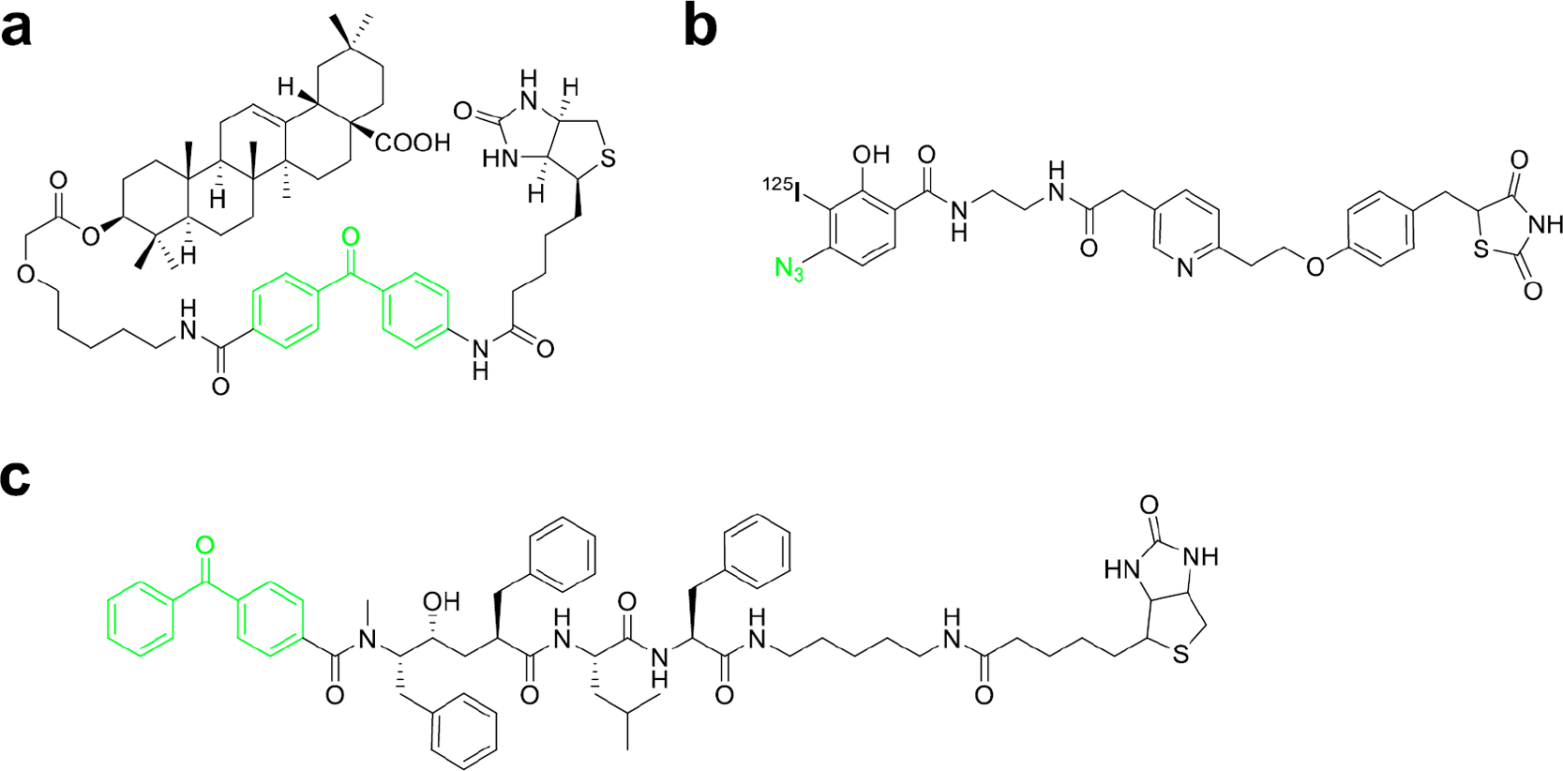
(a) Chimeric molecule designed to identify the target of oleanolic acid; (b) photoactivated-γ-secretase inhibitor in which the tag covalently label presenilin 1; (c) photoaffinity labeled palmytoil derivative directed to the peroxisomal β-oxidation enzyme. Shown in green are the photoreactive groups.

Other engineered chimeric structures have been adopted for tagging experiments. For example, a chimeric molecule to identify the target of oleanolic acid was prepared^176^ (Figure 20a), and a photoactivated γ-secretase inhibitor in which the tag covalently label presenilin 1 was developed^177^ (Figure 20b). Presenilin 1 belongs to the γ secretase complex and plays an important role in the generation of amyloid β (Aβ) from the amyloid precursor protein, and it is associated with the onset of Alzheimer disease. The novel photoaffinity labeled palmitoyl derivative (Figure 20c) is directed to the peroxisomal β-oxidation enzyme, a primary enzyme for fatty acid degradation.^178^

Recently a high number of PAL applications have been published, underlining the increasing importance of this method in drug discovery.

Other technologies are under development within the engagement technology field such as the carbene footprinting technology.^179,180^ An example is given by the differential protein footprinting approach that adopted an efficient photoactivated probe and used it in mass spectrometry to map the binding cleft of lysozyme, as well as between UPS5, a deubiquitinating enzyme, and a diubiquitin substrate.^179^

## 5 Conclusion

The development of new technologies and chemical biology strategies has largely stimulated medicinal chemists’ creativity to design molecules that could meet the challenges that a drug encounters from the delivery to the patient up to target binding.

This ambitious task was initially addressed using simple structures with chemicophysical properties suitable for cell membrane penetration. However, chemistry exploration and modular approaches led to the design of engineered constructs called chimeric molecules. Chimeras have been exploited in a wide range of applications, such as drug targeting and release, drug tracking and monitoring when tagged with fluorescent probes, target engagement, and mechanism of action clarification. Recently, engineered systems in which both compounds/drugs and proteins are chemically modified to give more specific and less invasive assays have been developed. Crucial is the role of the linking fragment connecting the functional head with the tag. Starting from a disulfide and an ester, the first linkers were based on the early concept of prodrugs, in which a cleavable bond could easily release the bioactive compound. Application of this linker chemistry was promising; however, the use of these systems was hampered by the risk of low specificity. Improved engineered compounds were developed, and linkers were recognized as an essential tool for structure–activity relationship studies of chimeric compounds and for providing the requested reactivity to conjugate the head and the tag. From a linear structure, such as PEG and alkyl chains with reactive groups at the two edges, a “three-dimensional” decoration of the chain is taking place to address the biological requirements, as observed in some ADCs. Irrespective of application field, chimeric molecules and linkers are conceptually related and can be exploited also in fields different from those mentioned in the present Perspective, including biosensors, biomarkers, and molecular machine. Chimeras are being developed by teamwork of medicinal chemists and chemical biologists and represent formidable tools for targeted therapies and personalized medicine.

## Abbreviations Used

RME: receptor mediated endocytosis
ADC: antibody–drug conjugate
CPP: cell-penetrating peptide
THP: tumor homing peptide
mAbs: monoclonal antibodies
SDS–PAGE: sodium dodecyl sulfate–polyacrylamide gel electrophoresis
GST: glutathione
PROTAC: proteolysis targeting chimera
CRBN: cereblon
TBAF: tetra-*n*-butylammonium fluoride
TR-FRET: time-resolved Förster resonance energy transfer
BRET: bioluminescence resonance energy transfer
FLIM: fluorescence lifetime imaging microscopy
BET protein: bromodomain and extraterminal domain protein family
CDK9: cyclin dependent kinase 9.
